# Larval Exposure to Parasitic *Varroa destructor* Mites Triggers Specific Immune Responses in Different Honey Bee Castes and Species

**DOI:** 10.1016/j.mcpro.2022.100257

**Published:** 2022-06-13

**Authors:** Yu Fang, Abebe Jenberie Wubie, Mao Feng, Chuan Ma, Boris Baer, Jianke Li

**Affiliations:** 1 Institute of Apicultural Research/Key Laboratory of Pollinating Insect Biology, Ministry of Agriculture, Chinese Academy of Agricultural Sciences, Beijing, China; 2 Department of Animal Production and Technology, College of Agriculture and Environmental Sciences, Bahir Dar University, Bahir Dar, Ethiopia; 3 International Center of Insect Physiology and Ecology (ICIPE), Addis Ababa, Ethiopia; 4 Center for Integrative Bee Research (CIBER), Department of Entomology, University of California Riverside, Riverside, USA

**Keywords:** insect immunity, innate immunity, honey bee health, host–parasite interactions, evolutionary proteomics, GO, Gene Ontology, IAR-CAAS, Institute of Apicultural Research, Chinese Academy of Agricultural Sciences, MS, mass spectrometry, PPI, protein–protein interaction, qRT–PCR, quantitative RT–PCR, SPME, solid-phase microextraction

## Abstract

Innate immune systems are key defenses of animals and particularly important in species that lack the sophisticated adaptive immune systems as found in vertebrates. Here, we were interested to quantify variation in innate immune responses of insects in hosts that differ in their parasite susceptibility. To do this, we studied immune responses in honey bees, which can host a remarkable number of different parasites, which are major contributors of declining bee health and colony losses. The most significant parasite of honey bees is the mite *Varroa destructor*, which has infested the majority of global honey bee populations, and its control remains a major challenge for beekeepers. However, a number of nonmanaged honey bees seem able to control *Varroa* infections, for example, the Eastern honey bee *Apis ceranacerana* or the African honey bee *Apis mellifera scutellata*. These bees therefore make interesting study subjects to identify underlaying resistance traits, for example, by comparing them to more susceptible bee genotypes such as Western honey bees (*A. melliferaligustica*). We conducted a series of interlinked experiments and started with behavioral assays to compare the attractiveness of bee larvae to mites using different honey bee genotypes and castes. We found that 6-day-old larvae are always most attractive to mites, independently of genotype or castes. In a next step, we compared volatile profiles of the most attractive larvae to test whether they could be used by mites for host selection. We found that the abundance of volatile compounds differed between larval ages, but we also found significant differences between genotypes and castes. To further study the expected underlaying physiological differences between potentially resistant and susceptible host larvae, we compared the larval hemolymph proteomes of the three honey bee genotypes and two castes in response to mite exposure. We identified consistent upregulation of immune and stress-related genes in *Varroa*-exposed larvae, which differed between genotypes and castes. Tolerant honey bee castes and genotypes were characterized by stronger or more distinct immune esponses. In summary, we provide first insights into the complex involvement of the innate immune system of tolerant honey bees against mite infestations, which could be used for future breeding purposes.

## Highlights

• Honey bee larvae are most susceptible to mite infestations shortly before brood cell capping.
• Larval attractiveness to mites is independent of bee caste or genotype.
• Volatile compound abundance differed between genotypes, castes, and larval ages.
• Mite exposure induces specific immune and stress responses in different genotypes and castes.

## Graphical Abstract

**Figure:**
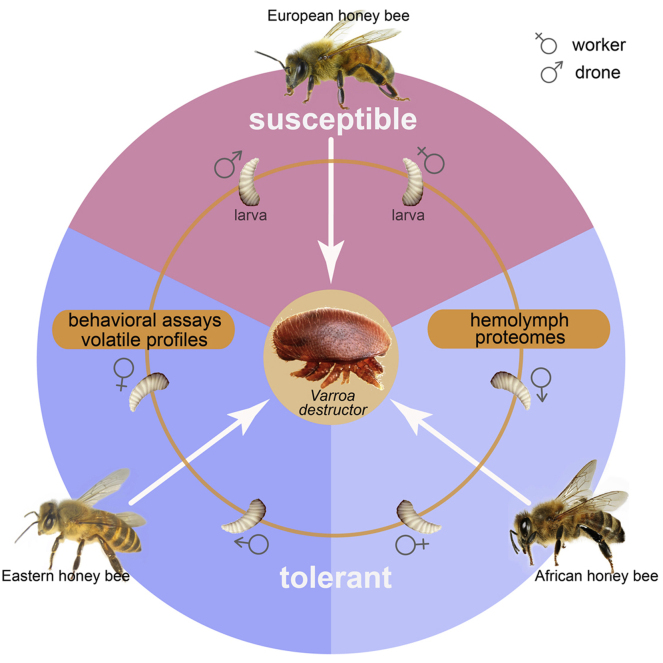

## In Brief

We used three honey bee genotypes and investigated their attractiveness and physiological responses to parasitic *Varroa destructor* mites. Mites preferred 6-day-old larvae, irrespective of their species/castes. Comparing larval hemolymph proteomes revealed physiological responses to mite exposure in all genotypes and castes, but tolerant larvae responded more pronounced by increasing the abundance of proteins linked to immune and stress responses. We provide key insights into the complex involvement of the honey bee immune system against mite infestations, which could be used for future breeding purposes.

The innate immune system is a key defense mechanism against parasites (1). It is also involved in related physiological activities such as wound healing (2) but can be costly for its host if it triggers self-harming autoimmune reactions (3). It has been studied intensively in the past, especially in insects that lack the adaptive immunity as present in vertebrates, and is therefore of particular importance for these animals to combat parasites and pathogens. Innate immunity can be induced but is assumed to lack the specificity and memory of adaptive immunity and is generally regarded as a first and more generic immune response to infection or parasite exposure. However, more recent research concluded that innate immunity is involved in a large number of different physiological processes (4, 5, 6, 7, 8) and is therefore more complex than originally anticipated. The use of systems biology now allows to not only unravel the full complexity of innate immune responses but also quantify genetic and phenotypic variation, the latter being key determinates of host tolerance as well as host–parasite coevolution.

Here, we studied the innate immune system in honey bees, which are of central importance for ecosystem stability and human food production (9, 10). They have been largely taken for granted, but substantial declines in managed (11) and wild (12) bee populations have been documented over recent years. Research into declining bee health has identified several contributing factors, including environmental changes such as climate change (13) and habitat loss (14), inferior beekeeping practices as well as the exposure of bees to agricultural pesticides (15), especially on sublethal levels (16). However, parasites and pathogens are well-documented key culprits responsible for declining bee health (17). This is especially true for Western honey bees (*Apis mellifera* *ligustica*), which are the globally most widespread managed pollinator and harbor an unusually large number of parasites (18). Their impact on bee health and colony performance depends on the individual parasite species and the presence of other environmental stressors such as pesticides (19), but parasites are well-documented triggers of colony collapses and population declines (20).

The globally most significant parasite of honey bees is the mite *Varroa destructor* (21). This parasite was able to switch hosts during the last century from its original host, the Eastern honey bee (*Apis cerana* *cerana*), to Western honey bees (*A. mellifera* *ligustica*) and African honey bees (*A. mellifera scutellata*) (22, 23). Because of the widespread use and transportation of managed Western honey bees, the parasite was able to spread globally and is now present on all bee-inhabiting continents except a few island populations (24). The detrimental impact of *Varroa* on honey bees is amplified because these mites act as vectors for other honey bee diseases such as viruses (25). *Varroa* mites are essentially brood parasites that infest and reproduce on developing larvae (26). To achieve this, mites need to successfully infest suitable larvae and escape expected immune responses of their hosts. This seems not always successful because mite reproductive success varies substantially, and failure to reproduce is common in mites, as up to 20% of mites are infertile after successfully invading larval cells (27). Mite infertility seems induced by host factors (28), but the underlaying molecular mechanisms of such tolerance traits of honey bees toward *Varroa* remains to be unraveled.

Some honey bees are known to naturally tolerate and survive *Varroa* infections. This has been documented for both Eastern (26) and African honey bees (29). Interestingly, the crossing between *Varroa*-tolerant African honey bees and susceptible Western honey bees resulted in a hybrid honey bee species known as Africanized honey bees. These bees also possess *Varroa* resistance traits (30), which must have been a key life history trait to promote their successful spread throughout the Americas (31). Given that Western honey bees are highly susceptible to *Varroa*, the tolerance and/or resistance traits reported in Africanized honey bees must therefore have originated from their African ancestors.

A number of studies investigated possible behavioral adaptations of *Varroa*-tolerant honey bees as defense mechanisms to control *Varroa* infections, known as social immunity (32). These include autogrooming behaviors (33) as well as *Varroa*-sensitive hygienic behaviors (27) where worker bees recognize and remove infested larvae or mites from other bees. Mite infections can also trigger swarming behavior (34), where bees relocate their colony and thereby suppress mite levels through the resulting interruption of the brood cycle and by leaving infected brood and mites behind. Despite these findings, *Varroa* resistance of honey bees remains poorly understood but is likely the result of multiple factors and not only based on social immunity. Whereas behavioral adaptations received substantial scientific interest (35, 36), much less work has been conducted to test for the possible involvement of the innate immune system in the recognition and defense against mites. Comparative studies of *Varroa*-tolerant *versus* susceptible honey bee genotypes therefore offer the opportunity to test for the possible involvement of the innate immune system in responses to *Varroa* infections. Recent work confirmed that components of the innate immune system are remarkably efficient to recognize and kill honey bee parasites such as the fungal pathogen *Nosema* (37, 38, 39). This work also developed the necessary methodologies to use systems biology approaches to unravel the metabolomic and proteomic mechanisms underlaying innate immunity traits in honey bees (40, 41, 42).

Here, we conducted a series of behavioral and molecular experiments with the aim to study the dynamics of both mite host choice and honey bee immune responses. We focused on a crucial point in the parasites’ life cycle, that is, the period when phoretic mites select host larvae and initiate their reproductive cycle. We started with a set of behavioral host choice experiments using male and worker larvae of African (*A. mellifera scutellate*), Eastern (*A. cerana cerana*), and Western (*A. mellifera ligustica*) honey bees to identify those larval life stages that are most susceptible to mite infestations. Our main aim of this study was to compare immune responses in different honey bees with documented differences in parasite tolerance, rather than to understand the evolution of disease resistance in different bee species. We will therefore refer to the three different types of bees used as different bee genotypes, because some represent different species (*e.g.*, Eastern *versus* Western honey bees), whereas others are different subspecies (Western *versus* African honey bees). We found that, irrespectively of bee genotype or castes, larval attractiveness to mites is maximal at an age of 6 days, which corresponds to the period shortly before brood cells are capped and mites become less susceptible to social immunity. We consequently compared the shared volatile profiles of least and most attractive larvae to see if they are key to attract mites to their host larvae. We found several volatile compounds in higher concentrations in more attractive larvae but also surprising variation between genotypes and castes. We therefore followed up with a comparative proteomic experiment that compared proteomes of most attractive larvae in response to mite exposure to nonexposed ones in all genotypes and castes. We found that larvae of all genotypes and castes respond with unique changes in protein expression to the presence of mites and provide empirical support for the idea of a complex involvement of the innate immune system of different honey bees against *Varroa*.

## Experimental Procedures

### Honey Bee Breeding and *Varroa* Mite Collection

All beekeeping equipment used for experiments were obtained from Henan Multi-Sweet Beekeeping Technology Co, Ltd. Chemical reagents used for experiments were of analytical grade and purchased from Sigma–Aldrich, unless specified otherwise. To unravel the behavioral and physiological interactions between the parasitic mite *V. destructor* and their African (*A. mellifera scutellata*), Eastern (*A. cerana cerana*), and Western (*A. mellifera ligustica*) honey bees hosts, we reared worker and drone larvae of specific ages using standardized apicultural practices (43). We kept six colonies each of Western (the queens were imported from Bologna, Italy) and Eastern honey bees at an apiary at the Institute of Apicultural Research, Chinese Academy of Agricultural Sciences (IAR-CAAS) in Beijing, China. Because African honey bees are not native to China, we set up a comparable apiary at the College of Agriculture and Environmental Sciences at Bahir Dar University in Ethiopia for experimental work using local colonies of *A. mellifera scutellata*. To standardize colony size, we initially set up six nucleus hives of all three genotypes that each contained four frames with full covered bees, three empty brood frames, and a single newly mated queen. All colonies were kept for 5 weeks prior to any experiments. All work conducted was approved by the IAR-CAAS Review Board.

We used a larval grafting tool to collect *V. destructor* mites by opening sealed brood. Our initial aim was to conduct experiments using mites collected from the same bee host genotypes. As might be expected, we found only very low or nondetectable levels of *Varroa* infections in our colonies of Eastern honey bees, confirming the previously reported elevated *Varroa* tolerance levels in the colonies we used for our experiments. We consequently used mites that we collected from Western honey bees for experiments in Eastern honey bees. We did not anticipate that this impacted our results and conclusions, because Eastern honey bees are the original host of *V. destructor* and are naturally infested with this parasite. Furthermore, our prime interest was to ultimately study host responses following mite exposure, so all mites and bees were furthermore sourced locally and from a geographic region where both bee species coexist. Consequently, we predicted both bee species to have been exposed to the mite genotypes we used for our experiments. We kept all mites in an incubator after collection at 34 °C prior to further experimental use.

### *In Vitro* Rearing of Honey Bee Larvae

For each bee genotype, we reared worker and drone larvae in the laboratory using methods described earlier (43). In brief, we caged mother queens onto a brood frame for 1 day to allow them to lay eggs. We collected first instar larvae at an age of 4 days and transferred them individually to single plastic wells using a 24-well plate containing 50 μl preheated (34 °C) larval food made from 50% fresh royal jelly, 6% d-glucose (w/v), 6% d-fructose (w/v), 1% yeast extract (w/v), and 37% double-distilled water. We kept larvae in an incubator at 34 °C and 85% relative humidity in the dark and checked them daily to clean their wells and provided them with fresh food ad libitum. Larval development was continuously monitored using an infrared video camera that we mounted inside the incubator.

### *Varroa* Host Choice Experiments

**Fig. 1:**
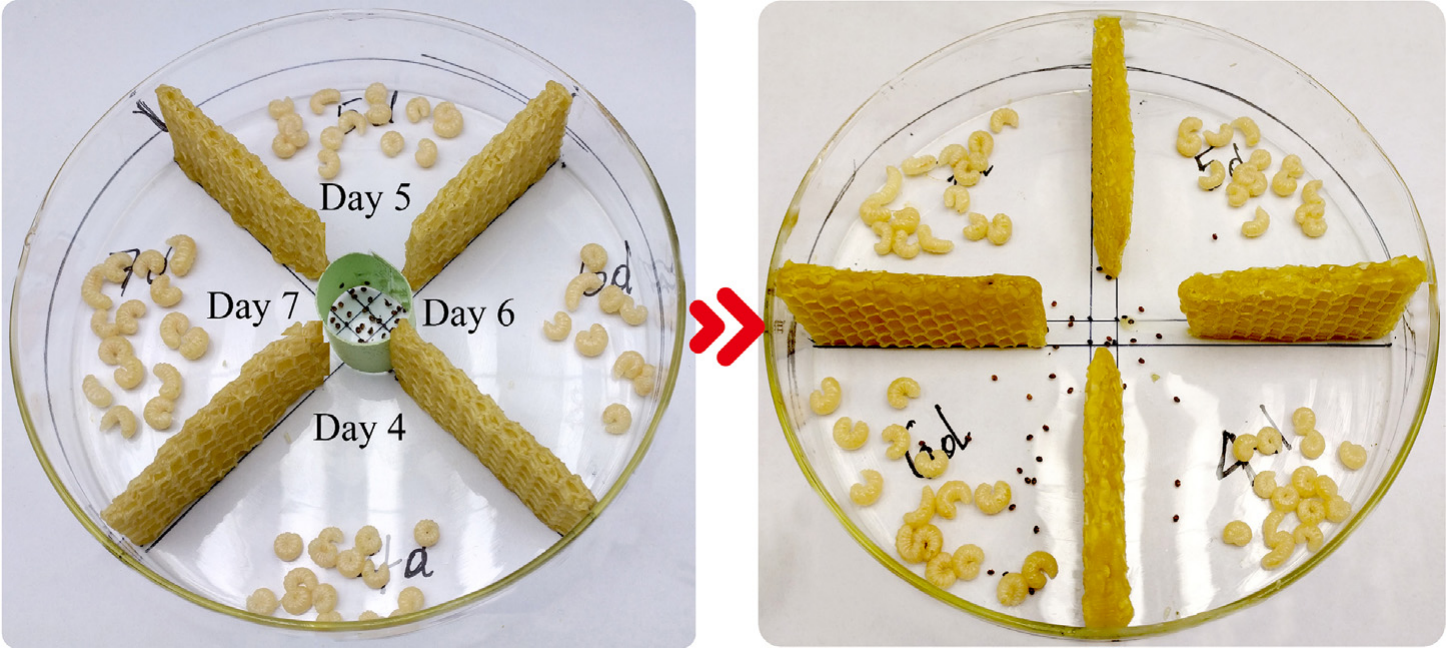
**Setup of *Varroa* choice experiments.** Twenty bee larvae at an age of 4, 5, 6, or 7 days each were placed in one of four petri dish quadrants separated by honey bee wax foundation. Thirty *Varroa* mites were added to the central area and separated by a cardboard tube. All petri dishes were transferred to a dark incubator at 34 °C and 75% relative humidity. The cardboard tube was removed after 15 min, allowing mites to freely move into quadrants and infest larvae. We quantified the number of mites on larvae every 15 min for a total of 2 h, resulting in a total of eight measurements per assay. *Varroa* choice experiments were conducted for each of the three honey bee genotypes (*Apis mellifera scutellata*, *Apis cerana cerana,* and *Apis mellifera ligustica*) and castes (drones or workers) using five replicates per assay resulting in a total sample size of 30 assays.

To quantify the attractiveness of different honey bee larvae to *Varroa* mites, we conducted choice experiments using methods developed earlier (44) by presenting male/worker larvae of different ages to mites. To do this, we used bee wax to separate a petri dish with a diameter of 150 mm into four equal quadrants leaving the central part with a diameter of approximately 10 mm undivided (Fig. 1). We then placed 20 bee larvae each at an age of 4, 5, 6, or 7 days in one of the four quadrants. Thirty *Varroa* mites were added to the central nondivided area of the petri dish. We used a cardboard tube to initially restrict the mites in the central area to allow mites to get used to their new environment (Fig. 1). We transferred petri dishes to an incubator at 34 °C and 75% relative humidity and kept them in the dark recording mite movements with an infrared camera. After 15 min, the cardboard tube was removed, which allowed mites to freely move into quadrants and infest larvae. We quantified the number of mites on larvae every 15 min for a total of 2 h, resulting in a total of eight measurements per assay. We conducted choice experiments for each of the three honey bee genotypes and castes (drones or workers) using five replicates per resulting in a total of 30 assays. To analyze the data, we used a generalized linear model with a negative binomial probability distribution and log link function, with the total number of mites found per larval age group as dependent variable, bee genotype, caste, and replication as independent factors, and larval age and observation time as covariates. We first conducted a full analysis and then removed all nonsignificant interaction terms stepwise. We also inspected video recordings for a qualitative analysis to identify possible behavioral responses of larvae in response to mite infestations.

### Volatile Profiles of Attractive Honey Bee Larvae

To test whether one or very few common specific larval volatiles explain the observed differences in *Varroa* mite host choice, we identified a set of emitted volatiles present in all larvae and compared them between male and worker larvae of Eastern and Western honey bees. We reared worker and drone larvae of both bee genotypes in the laboratory to an age of 4 and 6 days, the ages we found to be the least and most attractive to mites (see Results section). For each assay, we placed 15 larvae in a 20 ml headspace vial containing 3 μl of internal standard (*n*-heptadecane, 25 μg/ml in *n*-hexane) and kept them at 34 °C for 5 min before compound extraction. Emitted volatiles of individual larvae were collected using a solid-phase microextraction (SPME) fiber (DVB/CAR/PDMS; Supelco). To analyze samples, we used an autosampler GC-MS2010 system (Shimadzu Scientific Instruments) that was initially conditioned for 7 min inside the GC injector port at 250 °C. The SPME fiber was first inserted into the headspace vial and kept at 34 °C for 30 min to absorb volatiles and consequently transferred to the GC injector port and thermally desorbed at 265 °C for 1 min. We used a DB-5 column (30 m × 0.25 mm × 0.25 μm; Agilent) for compound separation. The oven temperature was initially set at 35 °C for 1 min and then gradually increased to 200 °C at a rate of 5 °C per min. The temperature was then increased to 250 °C at 15 °C/min and finally kept at 250 °C for 2 min. We used helium with a purity of >99.999% as carrier gas and at a constant flow of 1 ml/min. The ion source was set at 230 °C, ionization energy at 70 eV, and transmission line temperature at 270 °C. The solvent delay time was set to 3.5 min, and scanned mass ranges between 40 and 400 *m/z*. The SPME fiber was reconditioned in the GC injector port prior to further experimental use. We analyzed GC–MS data using the XCMS metabolomics platform (http://xcmsonline.scripps.edu/) for feature detection using the matched filter algorithm (45). For statistical analyses of individual compounds, the peak area was normalized to the internal standard. A mixture of C8–C30 *n*-alkanes dissolved in *n*-hexane was used at the same conditions as described previously to calculate retention indexes. Individual compounds were identified by comparing mass spectrometry (MS) fragmentation patterns and retention indices with those provided by the National Institute of Standards and Technology database (NIST, version 14). An empty headspace vial was used as a control to confirm that the compounds identified originated from the larvae. Volatile extractions and analyses were conducted using six replicates for each age group, caste, and both bee genotypes, resulting in a total of 96 samples that became available for analyses. We used ANOVA to test for significant differences in compound abundances between genotypes, castes, and ages.

### Proteomic Responses of Larvae to *Varroa* Exposure

To quantify physiological responses in the hemolymph of worker or male larvae following *Varroa* infestations, we exposed the most susceptible 6-day-old larvae of the three bee genotypes for 2 h to mites and compared their hemolymph proteomes to nonexposed control individuals. To do this, we collected 10 μl of hemolymph per larvae using a method described earlier (46) and pooled the hemolymph of 20 drone or 20 worker larvae at an age of 6 days. We collected six biological replicates per genotype, caste, and treatment (*Varroa* exposed *versus* control) resulting in a total of 72 samples originating from 1440 individuals that we used for protein identification and to compare their abundance among samples and treatments.

Extraction and digestion of hemolymph proteins was done using a previously established protocol (47). In brief, samples were homogenized on ice for 30 min using lysis buffer (8 M urea, 2 M thiourea, 4% CHAPS, 20 mM Tris base, 30 mM DTT) and centrifuged afterwards at 15,000*g* and 4 °C for 20 min. We added twice the volume of ice-cold acetone to each supernatant for protein precipitation and desalination and kept samples on ice for 30 min, followed by centrifugation at 15,000*g* and 4 °C for 20 min. The supernatants were discarded, and the protein pellets resuspended in 100 μl of 5 M urea and 400 μl of 40 mM NH_4_HCO_3_. Protein concentration was quantified in each sample using a Bradford assay. Proteins were reduced using 10 mM DTT for 60 min to prevent reformation of disulfide bonds, and iodoacetamide was added to a final concentration of 50 mM for alkalization and kept for 60 min in the dark.

Prior to MS–MS analyses, we used sequencing-grade modified trypsin (Promega) to digest protein samples at 37 °C overnight. Samples were resuspended in deionized water containing 0.2% formic acid, and a 10 μl subsample was analyzed with a nanoflow LC–MS/MS connected to an Orbitrap Q-Exactive mass spectrometer (Thermo Fisher Scientific) and coupled to an online reversed-phase chromatography Easy-nLC1000 (Thermo Fisher Scientific). Samples were loaded onto a trap column for 2 min (5.0 μm Aqua C18 beads, 2 cm long, 100 μm inner diameter fused silica; Thermo Fisher Scientific) in buffer A (0.1% acetic acid) and at a flow rate of 5 μl/min. Peptides were separated by analytical columns (20 cm long, 75 μm inner diameter fused silica trap column filing with 3.0 μm Aqua C18 beads; Thermo Fisher Scientific) using a 120 min gradient.

Peptide separation was performed using a linear acetonitrile gradient increase from 8% to 30% and 0.1% formic acid for a duration of 105 min at 600 nl/min. Any eluting peptides were injected into a Q-Exactive mass spectrometer *via* electrospray ionization. MS and MS/MS spectra were collected in data-dependent mode using the following experimental setup: We started with a full scan (resolution 70,000 at 400 *m/z*; 300–1800 *m/z*), followed by scans on the top 20 peptide subsamples. We used high-energy dissociation in the linear ion trap mass spectrometer at a resolution of 17,500, an isolation window of 2 *m/z*, and a normalized collision energy of 27. We furthermore used dynamic exclusion (charge exclusion: unassigned 1, >8; peptide match: preferred; exclude isotopes: on; and dynamic exclusion: 10 s). All MS/MS data were acquired in RAW format using Xcalibur (version 2.2; Thermo Fisher Scientific).

MS/MS data were analyzed using PEAKS DB (version 8.5; Bioinformatics Solutions, Inc) for protein identification by searching against the *A. mellifera* protein database (downloaded from National Center for Biotechnology Information in April 2017 together with common contaminants, resulting in a total of 22,575 entries). Parameters set for PEAKS search were as follows: precursor ion and MS/MS tolerances: 20 ppm and 0.05 Da; enzyme specificity: trypsin; maximum missed cleavages: two; fixed modification: carbamidomethyl (C, +57.02 Da); and variable modification: oxidation (M, +15.99 Da). The false discovery rate was set to ≤1% for both the peptide and protein levels. Only a protein with at least two unique peptides was considered to be identified.

To test for significant differences in protein abundance between *Varroa*-exposed and *Varroa*-nonexposed bees, data were analyzed using the label-free PEAKS Q quantitation module. Feature detection was performed separately in each of the samples using expectation-maximization algorithm. Same peptide features from different samples were aligned using a high-performance retention time alignment algorithm (48). Peptide and protein abundance were considered as being significantly different between samples if *p* values were <0.05 and fold changes >2. Hierarchical clustering was performed to group the expressional profile of differentially abundant proteins in different samples using uncentered Pearson correlation and average linkage in PEAKS DB software.

### Bioinformatic Analysis

To unravel the physiological functions of those proteins that are differentially expressed in *Varroa*-exposed bees compared with nonexposed controls, we obtained the unique identifier GI numbers of corresponding proteins and used them as an input for ClueGO, version 2.1.7 (INSERM, AVENIR Team, Integrative Cancer Immunology) for a functional category and pathway enrichment analyses, using the available Cytoscape plug-in (http://apps.cytoscape.org/apps/cluego). This allowed us to test for significantly enriched functional Gene Ontology (GO) categories and pathways in biological processes. To correct for multiple testing, we adjusted the significance levels using the Benjamini–Hochberg procedure (49). The nodes in functionally grouped networks were linked on the basis of their kappa score level (0.4) in ClueGO. GO tree levels were ranging from level 3 to 8, and default parameters were used for GO term restrictions (#genes and %gene cover). Hierarchical cluster analysis and principal component analysis were also employed using the Perseus software (version 1.6.1) package (Max Planck Institute of Biochemistry), the property of Creative Commons Attribution 4.0 International. To gain additional insight into possible functional interactions among those proteins that we found to be differentially expressed between *Varroa*-exposed *versus* nonexposed controls, we conducted a protein–protein interaction (PPI) network analyses using GeneMANIA (University of Toronto) (50). Because of a lack of detailed information about the function of these proteins in honey bees, we performed this analysis using *Drosophila* accession IDs that were based on sequence homology using BLAST (https://blast.ncbi.nlm.nih.gov/Blast.cgi). This allowed us to construct a PPI map, where node proteins were clustered according to the GO annotation and networks of predicted, genetic, and physical interactions were enabled. The final network was visualized in Cytoscape (Cytoscape Consortium).

### Validation of the Differentially Expressed Proteins by Real-Time Quantitative PCR

TRIzol reagent (Invitrogen) was used to extract total RNA from the challenged and nonchallenged drone and worker groups of African, Eastern, and Western honey bees to generate complementary DNA using Reverse Transcriptase kit reagents (Transgen), according to the manufacturer’s instructions. We selected several key node proteins selected from our PPI network analysis and confirmed increased transcription activity after mite exposure using quantitative RT–PCR (qRT–PCR). To do this, we used the four immune proteins peroxiredoxin 1 (Prx1), thioredoxin-2 (Trx2), ferritin heavy polypeptide-17 (FHP17), phospholipid hydroperoxide glutathione peroxidase (Gtpx), whereas elongation factor 1-α (EF1α) and malate dehydrogenase (Md) were used as a control, and ribosomal protein S18 was used as reference gene to normalize data. The primer pairs are provided in supplemental Table S1. Briefly, 2 μl of complementary DNA was quantified in duplicate for each sample using LightCycler 480 SYBR Green on a LightCycler 480. Cycling conditions were 15 min at 95 °C, 45 cycles of (15 s at 94 °C, 25 s at 55 °C, and 20 s at 72 °C). Melt curve cycles immediately followed 5 s at 95 °C, 1 min at 65 °C, and then gradual temperature rise to 97 °C at a rate of 0.11 °C/s followed by 30 s at 40 °C. Data are displayed as fold change above proliferative condition mRNA level using 2^−△△Ct^ values.

### Experimental Design and Statistical Rationale

For the mite choice experiments, 20 larvae of identical age were used per age class (4, 5, 6, or 7 days old). Five biological replicates were conducted, and choice experiments were repeated for each bee genotype and caste, resulting in a total of 30 assays that were conducted. Using the same experimental design, a total of 30 GC–MS assays were conducted (5 biological replicates per assay using 3 bee genotypes and 2 castes) to test whether specific volatiles explain the attractiveness of bee larvae toward mites. For the hemolymph proteome comparisons, we analyzed six biological replicates per bee genotype and caste, resulting in a total of 72 samples that became available for MS–MS analyses to test for proteomic differenes between mite-exposed *versus* nonexposed larvae. Expression levels of key proteins were quantified using real-time qPCR, which were done using three biological replicates for each gene of interest. Statistical analysis was performed using SPSS (version 25) for Windows (IBM Corp). For comparison of two groups, an unpaired Student’s *t* test was performed, whereas one-way ANOVA tests followed by Tukey’s multiple comparison tests or Chi-square test were performed for comparison of more than two groups.

## Results

### Larval Susceptibility to *Varroa* Mites

**Fig. 2:**
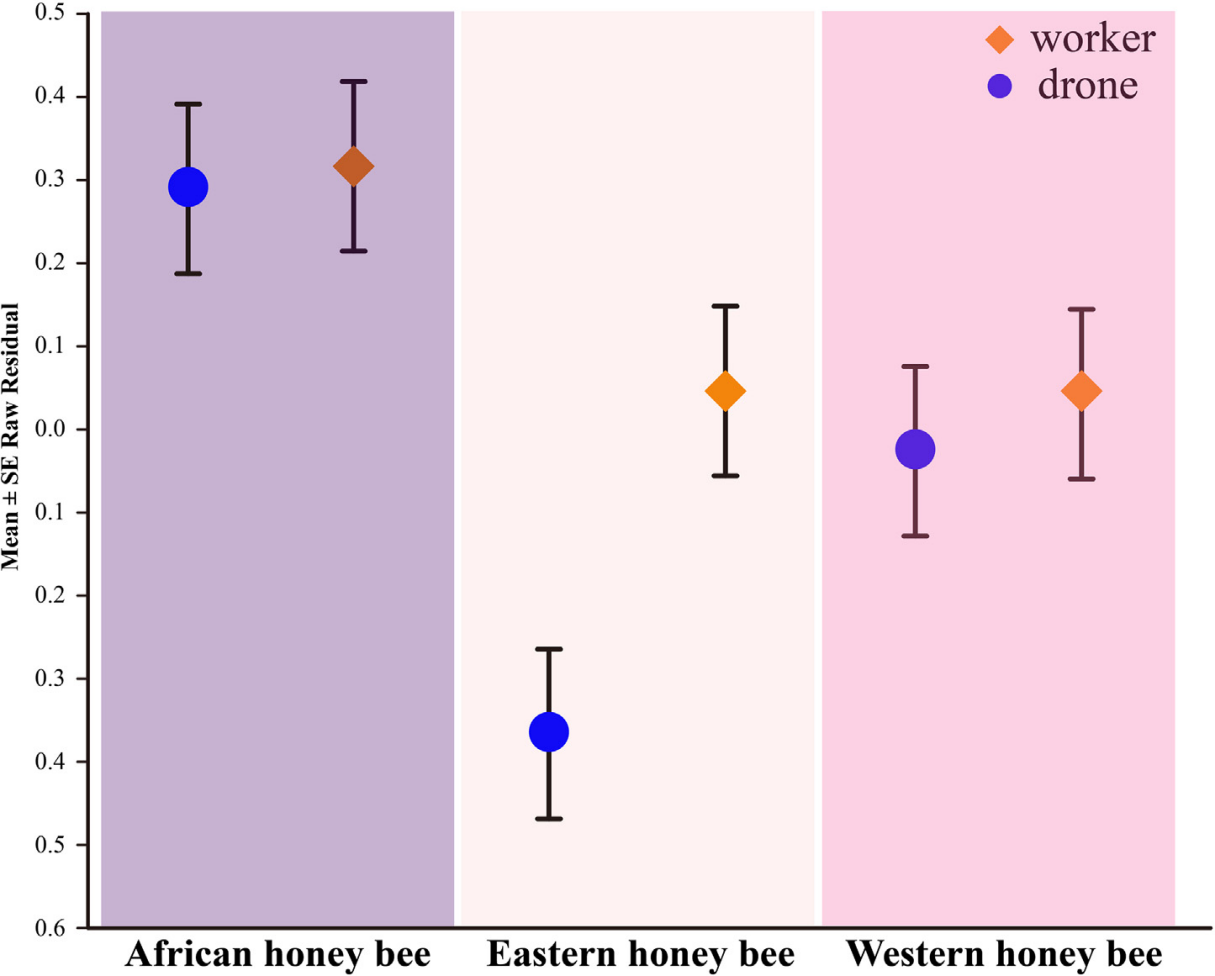
**Residual mite loads in larvae of Eastern (*Apis cerana cerana*), Western (*Apis mellifera ligustica*), and African (*Apis mellifera scutellate*) honey bees.** Worker larvae (*orange diamonds*) had higher mite loads compared with drones (*blue circles*) in Eastern honey bees, an effect that was not observed in Western and African honey bees.

**Fig. 3:**
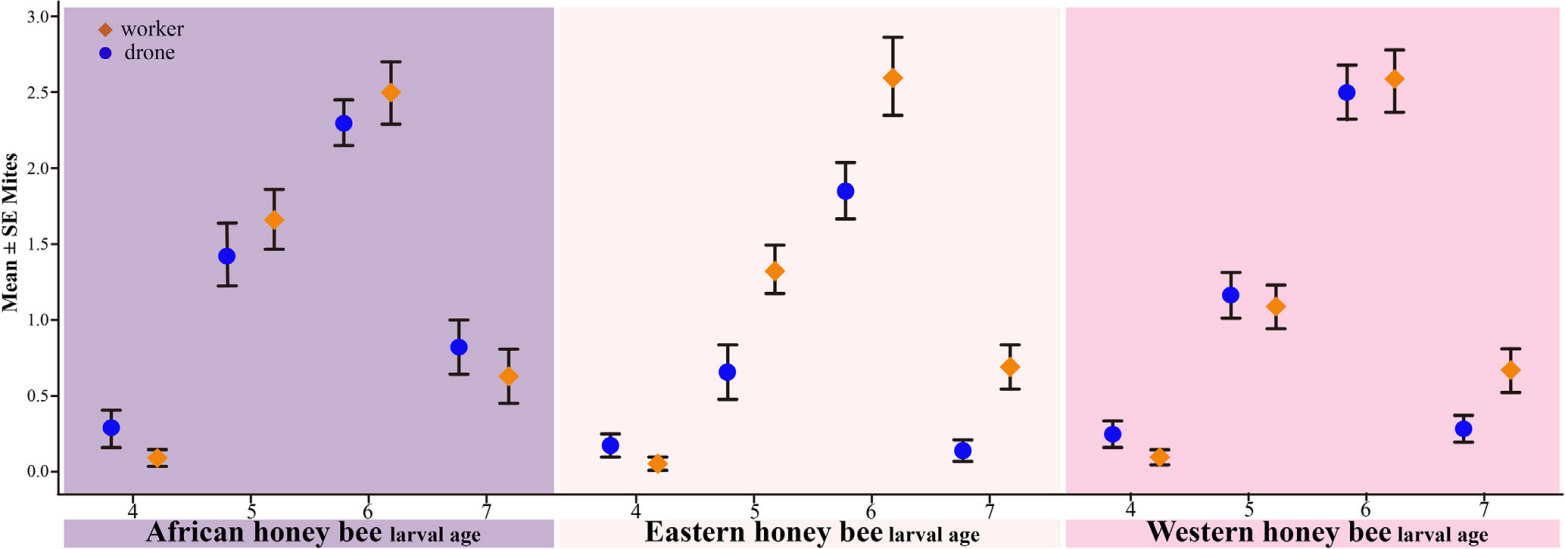
**The effect of larval age on *Varroa* host choice in Eastern (*Apis cerana cerana*), Western (*Apis mellifera ligustica*), and African (*Apis mellifera scutellate*)honey bees for both drones (*blue circles*) as well as workers (*orange diamonds*)**.

When we analyzed our behavioral observations of mite host choice, we found that mites were not attracted to larvae during the first 45 min of our observation period, irrespectively of host larval genotypes, ages, or castes. The inspection of our video recordings revealed that mites were behaviorally active during this period, but they did not move toward or onto individual bee larvae. We therefore conducted all statistical analyses of larval mite infestations using data we obtained 60, 75, 90, 105 and 120 min after the start of the trials. Mite loads on larvae increased over time (*p* < 0.001, see supplemental Table S2 for full statistical details), and we found a significant genotype × caste interaction term (*p* = 0.041, supplemental Table S2) indicating that mite infestations differed between male and worker larvae, but this was not consistent between the different bee genotypes. Mites were more attracted to worker compared to drone larvae in Eastern honey bees, but no such caste preference was observed in Western or African bees (Figs. 2 and S1). Larval age was a significant predictor of mite infestations, irrespective of genotype or caste and increased with larval age to maximal mite intensities in 6-day-old larvae, followed by a decrease in infection intensities in the 7-day-old larvae (Fig. 3). Because we found no significant differences between our biological replicates (*p* = 0.595, supplemental Table S2), we concluded that our observations were reproducible and consistent.

### Genotype and Caste Differences in Volatile Profiles of Most and Least Susceptible Honey Bee Larvae

To test whether specific volatiles explain the attractiveness of larvae toward mites, irrespective of genotype or caste, we compared the volatile profiles of Eastern and Western honey bee males and workers. We used larvae at an age of 4 days, which corresponds to the age they were the least attractive to mites (Fig. 3) and 6 days, when we found them to be most attractive to mites. We conducted a total of 30 GC–MS runs and identified a total of 15 volatiles that were present in all samples analyzed. This common set of volatiles included linear and branched-chain hydrocarbons containing 4 to 18 carbon atoms and shorter straight-chain hydrocarbons (supplemental Table S3). Quantifiable amounts became available for 2-hydroxy-3-butanone, 2-hexanone, 2-hexanol, pentanoic acid, 2-methyl-3-heptanone, beta-ocimene, methyl benzoate, nonanal, two different ocimene isomers, 2-decanone, heptadecene, ethyl tetradecanoate, methyl hexadecanoate, and ethyl palmitate (supplemental Table S4). Overall, we found that the genotype × caste interaction terms were always significant (supplemental Table S5) except for 2-hexanone. This implied that the abundance of these volatiles differed between male and worker larvae, but these caste differences were not consistent between the different bee genotypes. We found significantly higher concentrations of seven volatile compounds in 6-day-old larvae (supplemental Table S5), including all ocimene isoforms, ethyl palmitate, ethyl tetradecanoate, heptadecane, methyl benzoate, and methyl hexadecanoate.

We concluded from these experiments that age-related differences in volatile emission are substantially more variable between bee genotypes, castes, and age cohorts than we initially expected given the small group of shared volatiles as well as the remarkable variation in abundance between castes and genotypes. Although differences in volatile profiles could indeed be used by mites during host selection, the significant genotype × caste interaction terms implied substantial differences in the underlaying physiological responses of larvae depending on their genotype and caste. Given that they must result from differences in biochemical processes, we conducted a follow-up proteomic analysis to compare protein profiles and abundances between the different honey bee genotypes and castes in response to *Varroa* exposure. This allowed us to quantify a more systemic response of larvae to mite infestations including for example the upregulation of proteins specifically related to immune and/or stress responses.

### Comparative Proteomics of Larval Hemolymph

For each genotype and caste, we analyzed six biological replicates for both, *Varroa*-exposed larvae as well as nonexposed controls, resulting in a total of 72 samples. All mass spectrometral data have been uploaded at the ProteomeXchange Consortium (http://proteomecentral.proteomexchange.org) *via* the iProX partner repository (51) with the dataset identifier PXD029875. Overall Pearson’s correlation coefficients within replicates ranged from 0.9 to 0.97 (supplemental Fig. S2), indicating a high level of technical and biological reproducibility within our samples. Below, we start with a general comparison of the larval proteomes that we identified in the different genotypes and castes. In a next step, we then focus on the proteomic changes that we found in *Varroa*-exposed larvae compared to non-exposed controls and focus specifically on proteins and/or protein networks that we found to be upregulated in response to parasite exposure.

#### Comparative Hemolymph Proteomics of African, Eastern, and Western Honey Bees Not Exposed to Mites

**Fig. 4:**
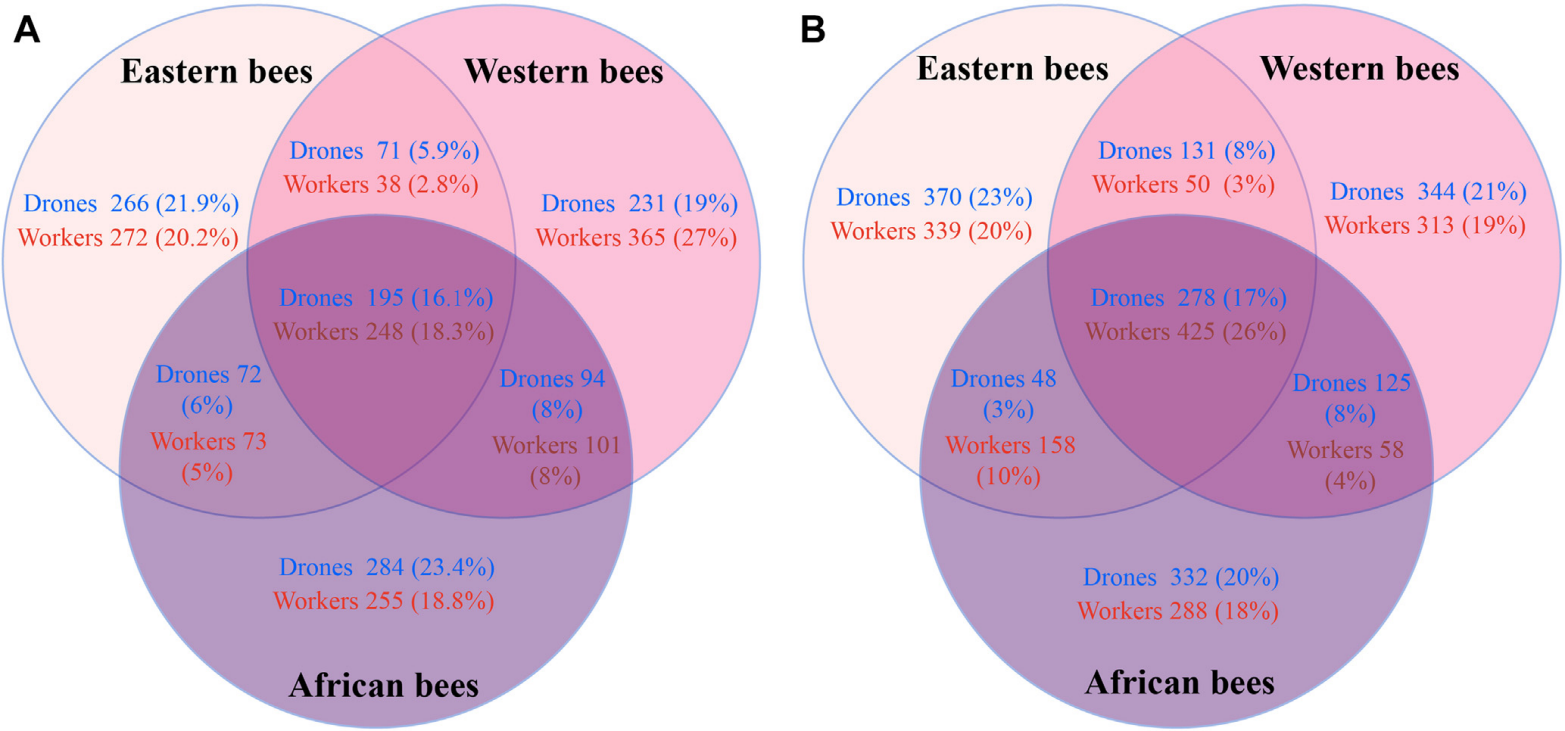
**A summary of specific and shared larval hemolymph proteins of workers and drones inAfrican (*****Apis mellifera scutellata*****),Eastern(*Apis cerana cerana*),andWestern(*Apis mellifera ligustica*)honey bees.***A*, summarizes findings for the bee larvae that were not exposed to mites; the numbers refer to individual proteins as listed in supplemental Tables S6–S8 for workers and supplemental Tables S9–S11 for drones. *B*, provides the number of proteins in larvae exposed to *Varroa* mites; for more information, see supplemental Tables S16–S18 for workers and supplemental Tables S19–S21 for drones.

**Fig. 5:**
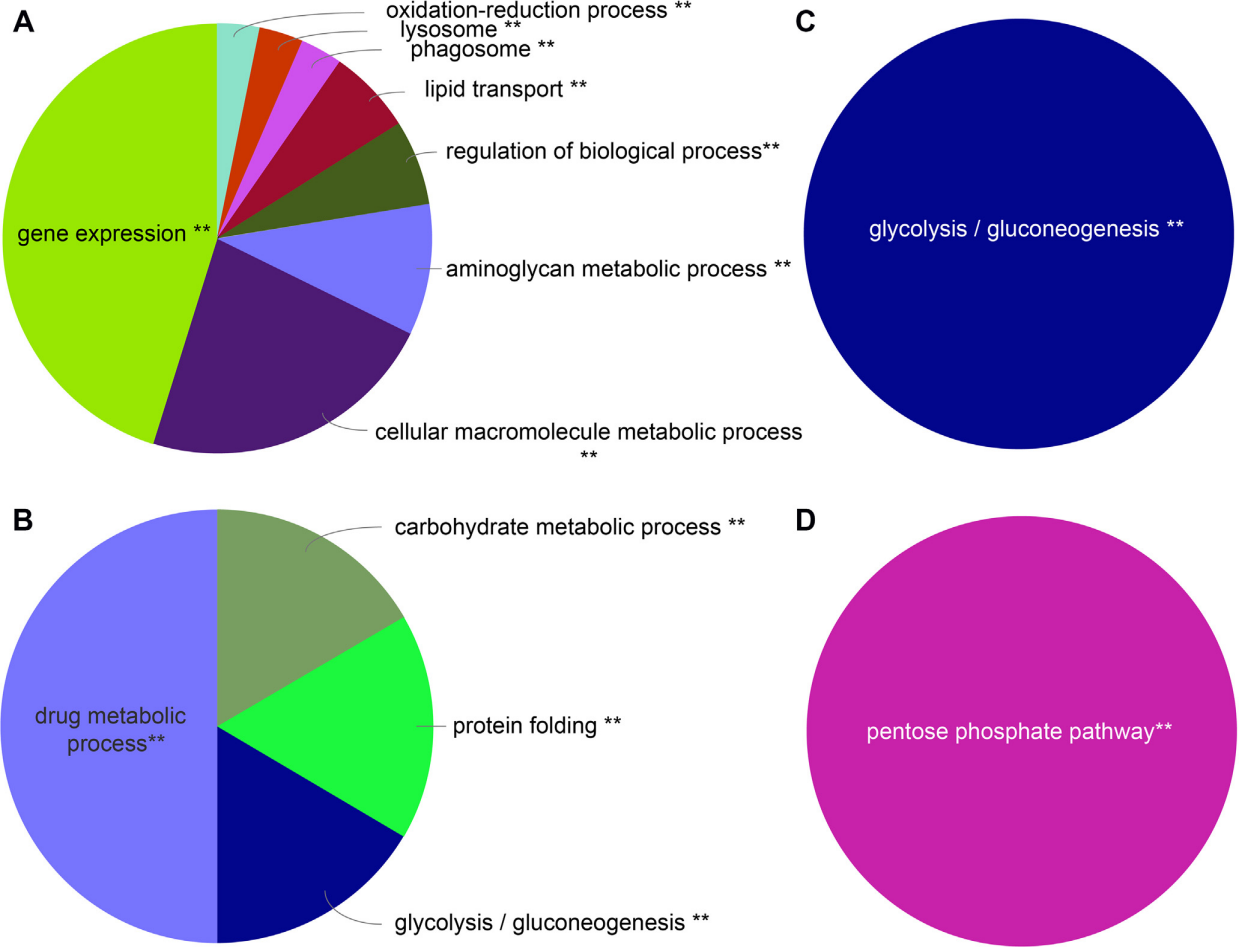
**Qualitative comparison of identifiedhemolymph proteins in honey bee worker larvae that were not exposed to*****Varroa*****mitesofEastern honey bee (*Apis cerana cerana*),Western honey bee (*Apis mellifera linguistica*), andAfrican honey bee (*Apis mellifera scutellata*)*.*** Identified proteins were analyzed using ClueGO to identify functional classes and pathways that are significantly enriched within a sample of interest. Chart A shows the significantly enriched functional classes and pathways shared among all nonchallenged honey bee larvae. Charts B, C, and D represent enriched functional groups and pathways for each of the three genotypes in *Varroa*-nonchallenged Eastern, Western, and African honey bee workers, respectively. *Asterisks* indicate significance levels; for more information, see supplemental Tables S15.

To compare the larval proteomes between males and workers of the three different bee genotypes, we first compared the 36 samples from the non–*Varroa*-exposed larvae. A summary of the number of proteins that we identified in each genotype and caste is provided in Figure 4*A* (supplemental Tables S6–S11 for a complete list of these proteins). We identified a total of 1940 different hemolymph proteins, 1354 in workers and 1213 in drones (see Fig. 4*A* for an overview of the number of proteins identified in each genotype and caste). The total number of proteins was comparable between the three genotypes, being 1027 proteins in African, 929 in Eastern, and 1079 proteins in Western honey bees but was somewhat higher in workers (supplemental Tables S6–S8) compared with males (supplemental Tables S9–S11). When we compared these proteomes, we found that although we identified comparable total numbers of proteins, the hemolymph of nonchallenged larvae of the three genotypes and two castes shares only a very small set of common proteins, 18.3% in workers (248 proteins) and 16.1% in drones (195 proteins, Fig. 4*A*). Within this group of proteins, the ClueGO software identified eight different functional groups that were significantly enriched in workers and involved in gene expression, oxidation–reduction process, lysosome, phagosome, lipid transport, regulation of biological process, aminoglycan metabolic process, and cellular macromolecule metabolic process (Fig. 5*A*). When we repeated the analysis for drones, we found some of the same pathways to be significantly enriched, such as lipid transport, aminoglycan metabolic process, and phagosome, but other pathways were drone specific such as carbohydrate metabolism, protein processing in endoplasmic reticulum, and glycolysis/gluconeogenesis (supplemental Fig. S3).

When we analyzed the nonshared proteins, we found that they were remarkably specific to the individual groups of larvae investigated. A substantial number of proteins were genotype specific, as we found 255, 272, and 365 proteins that were unique to the hemolymph proteomes of African, Eastern, and Western honey bee workers (Fig. 4*A*). The number of proteins only detected in Eastern honey bee workers was significantly enriched in several functional groups, such as carbohydrate metabolism, protein folding, glycolysis/glyconeogenesis, and drug metabolic processes (supplemental Fig. S5*B*). In Western and African honey bee workers, we found a significant enrichment of proteins involved in glycolysis/gluconeogenesis (supplemental Fig. S5*C*) and pentose phosphate pathway (supplemental Fig. S5*D*). We found comparable results in drone larvae, where 284, 266, and 231 proteins were unique to African, Eastern, and Western honey bee drones (Fig. 4*B*). In larvae of Western honey bee drones, we found that proteins involved in pentose phosphate and galactose metabolism were significantly enriched in Eastern drone larvae (supplemental Fig. S3*B*). Pathways responsible for cytoskeleton organization processes and pentose and glucuronate interconversions were enriched compared with the other two genotypes (supplemental Fig. S3*C*), whereas African bee larvae were characterized by an enrichment of pyridine nucleotide metabolic processes, glycan degradation, lysosome, and extracellular matrix–receptor interaction pathways (supplemental Fig. S3*D*). We concluded from these analyses that the nonshared group of proteins have distinct physiological activities both on the genotype and caste level, as they are part of very different biochemical pathways.

#### Protein Abundance Between Different Honey Bee Genotypes and Castes

**Fig. 6:**
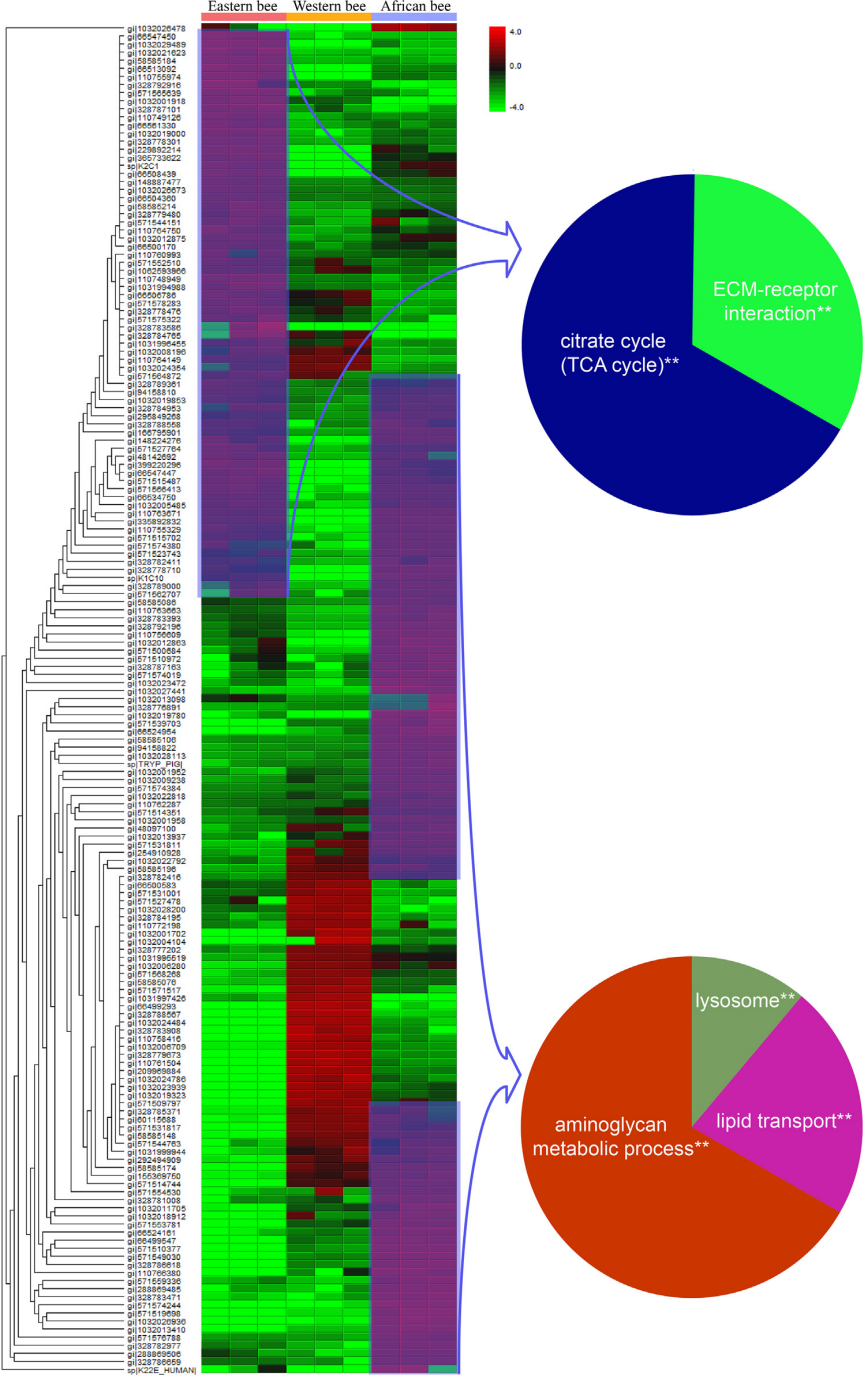
**Hierarchical cluster and pathway enrichment analysis of a quantitative hemolymph comparison innon-mite exposed-honey bee worker larvae ofAfrican (Ams: *Apis mellifera scutellate*), Eastern (Acc:*Apis cerana cerana*),andWestern (Aml*Apis mellifera linguistica*)honey bees.** Hierarchical cluster analysis was conducted using the Perseus software, version 1.6.1, package. Cytoscape plug-in ClueGO, version 2.1.7 (http://apps.cytoscape.org/apps/cluego) was used for pathway enrichment analyses. This allowed to test for significantly enriched KEGG pathways. To correct for multiple testing, we adjusted significance levels using the Benjamini–Hochberg procedure. KEGG, Kyoto Encyclopedia of Genes and Genomes.

Apart from analyzing proteome composition, we were also able to statistically compare the abundance of shared proteins in the different samples studied. We found that 161 proteins in workers (supplemental Table S12 and Fig. 6) and 114 in males (supplemental Table S13 and supplemental Fig. S4) differed significantly in their expression levels between larvae of the three nonchallenged honey bee genotypes. We found that 75, 48, and 38 proteins were significantly more abundant in African, Eastern, and Western workers (supplemental Tables S12 and S14, Fig. 6); whereas 52, 20, and 42 proteins were more abundant in drones (supplemental Tables S13, S14 and supplemental Fig. S4). The upregulated proteins in Eastern honey bee workers are part of the tricarboxylic acid cycle and extracellular matrix–receptor interaction pathways (Fig. 6). In African honey bee workers, proteins of higher abundance are involved in the lysosome pathway and aminoglycan metabolic processes (Fig. 6). We also found that higher concentration of proteins involved in lipid transport were found in African honey bees (supplemental Table S15 and Fig. 6). This latter finding was interesting because these functional groups are known from previous work to be involved in chemical communication between mites and bees (52). Interestingly, we found no biological process or pathway that was significantly upregulated in Western honey bee larvae. When we repeated this analysis for the three drone proteomes, we found that proteins involved in ascorbate and aldarate metabolic, glycolysis/gluconeogenesis, pentose phosphate pathway, arginine and proline metabolism, and β-alanine metabolism were present in higher abundance in nonchallenged Eastern drones (supplemental Fig. S4). African honey bee drone proteins involved in actin filament organization process were more abundant (supplemental Fig. S4). Similar to the results we found for workers, no significantly enriched functional classes or pathways were detected in Western honey bee drones.

#### Proteomic Changes in Larval Hemolymph in Response to *Varroa* Exposure

We used the full dataset of all 72 samples to conduct a comparative analysis to identify proteomic differences between mite-exposed *versus* nonexposed honey bee larvae. We identified 929, 972, and 846 proteins in *Varroa*-exposed African (supplemental Table S18), Eastern (supplemental Table S16), and Western (supplemental Table S17) honey bee workers, 425 (26.1%) of these proteins were shared among all species (Fig. 4*B*). We found a significant enrichment of shared proteins for a number of physiological processes, such as nucleic acid metabolism, nucleobase-containing compound biosynthesis, cellular macromolecule metabolism, pyridine-containing compound metabolism, lipid and transmembrane transport, phagosome, pentose and glucuronate interconversions, and regulation of cellular processes (supplemental Fig. S5*A*). When we analyzed the group of uniquely expressed proteins in African (288), Eastern (339), and Western (313) worker larvae (Fig. 4), we found enriched proteins to be part of five, seven, and four biological processes and pathways (supplemental Fig. S4, *B*–*D*). Pathways related to detoxification were significantly enriched in African and Eastern workers, but this was not the case in Western honey bee workers. For drone honey bees, the 278 (17.1%) shared proteins (Fig. 4*B*) were enriched in nine functional groups and pathways, including glutathione metabolism process, folate biosynthesis, protein folding, oxidation reduction process, glycolysis/gluconeogenesis, pentose phosphate pathway, arginine and proline metabolism, regulation of biological process, and cellular macromolecule metabolic process (supplemental Fig. S6*A*).

When we analyzed those proteins that were genotype specific for African (332, 20.4%), Eastern (370, 22.7%), and Western (344, 21.1%) honey bee drones (Fig. 4*B* and supplemental Tables S19–S21), we found significant enrichments for one, five, and five biological process(es) (supplemental Fig. S6). These included pathways related to immunity or stress response such as lipid transport and detoxification in Eastern, lipid transport in Western, and signal transduction in African drones (supplemental Fig. S6, *B*–*D*).

**Fig. 7:**
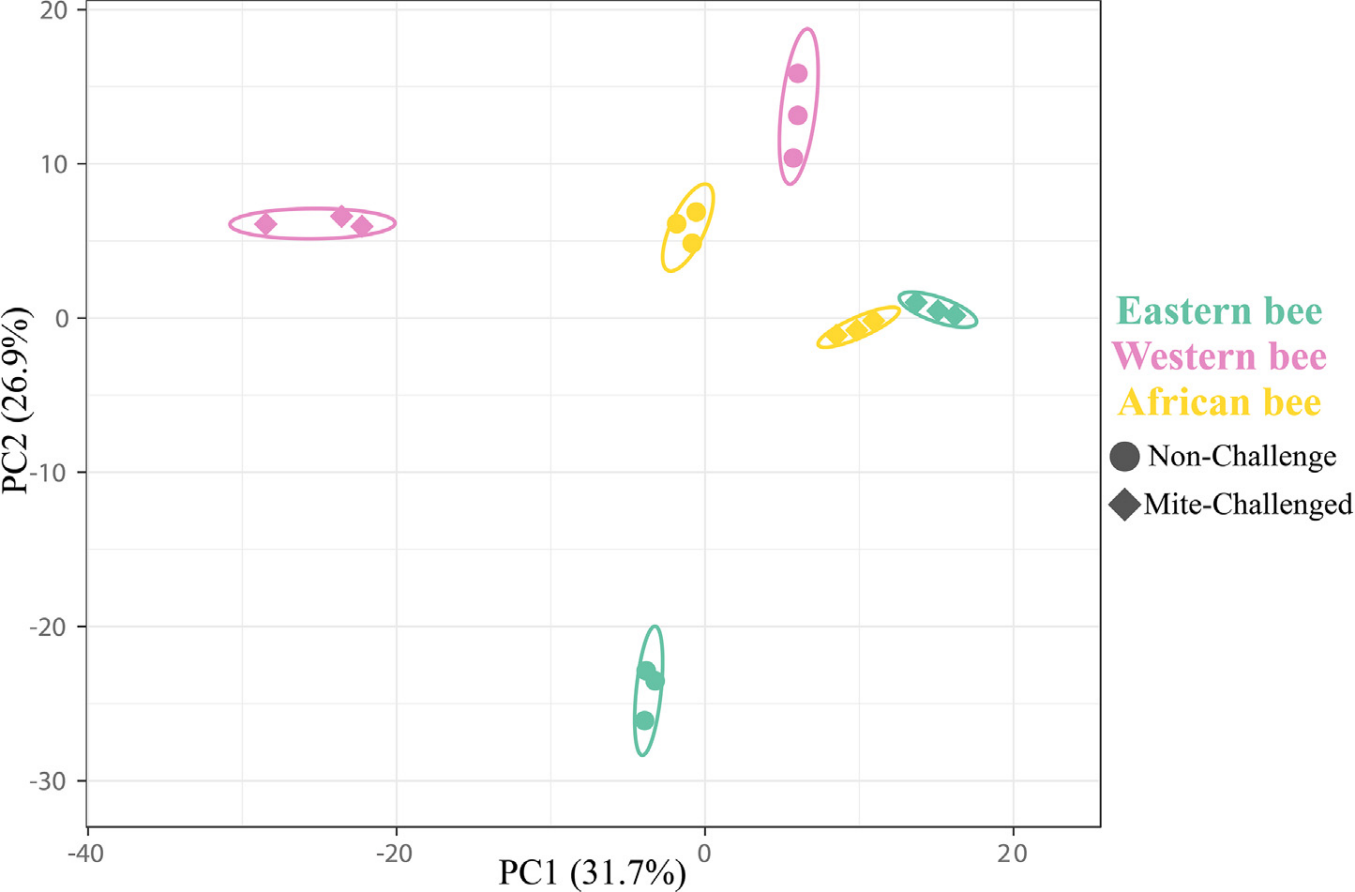
**Principal component analysis of identified hemolymph proteins in *Varroa* nonchallenged and challenged honey bee worker larvae ofEastern(*Apis cerana cerana*),Western(*Apis mellifera linguistica*), andAfrican (*Apis mellifera scutellate*)honey bees**.

**Fig. 8:**
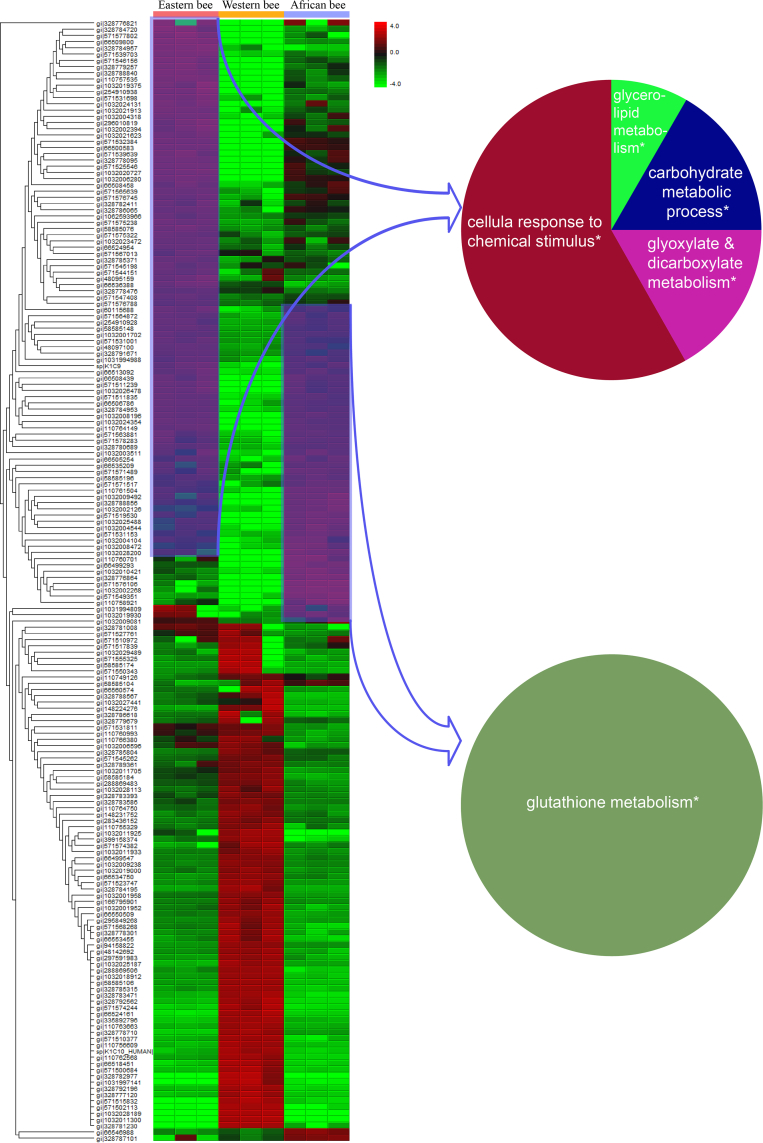
**Hierarchical cluster and pathway enrichment analysis of a quantitative hemolymph proteome comparison in mite-challenged honey bee worker larvae ofEastern (Acc:*Apis cerana cerana*),Western(Aml:*Apis mellifera linguistica*), andAfrican(Ams:*Apis mellifera scutellate*)honey bees.** Hierarchical cluster analysis was employed using the Perseus software, version 1.6.1 package. Cytoscape plug-in ClueGO, version 2.1.7 (http://apps.cytoscape.org/apps/cluego) was using for pathway enrichment analyses. This allowed us to test for significantly enriched KEGG pathways. To correct for multiple testing, we adjusted the significance levels using the Benjamini–Hochberg procedure. KEGG, Kyoto Encyclopedia of Genes and Genomes.

In a final step, we compared protein abundances using principal component analyses. We found that protein abundances of *Varroa*-challenged African and Eastern honey bee workers were more similar to each other compared with Western honey bee workers (Figs. 7 and S7). We found that 55, 73, and 55 proteins were differentially expressed in mite-exposed African, Eastern, and Western, and African honey bee worker larvae, respectively (supplemental Table S22). These proteins were significantly enriched for four functional groups in Eastern (Fig. 8), one functional group in African, and none in *Varroa*-susceptible Western bees (Fig. 8). Proteins involved in cellular responses to chemical stimuli were enriched in mite-challenged Eastern honey bee workers, whereas proteins linked to glutathione metabolism were enriched in African honey bees.

When we analyzed those differentially expressed proteins that were upregulated in mite-exposed larvae in drones, we found 41, 56, and 35 proteins in significantly higher abundances in African, Eastern, and Western honey bees (supplemental Tables S23 and S14). Pathways related to glycolysis/gluconeogenesis, pentose phosphate pathway, and aminoglycan metabolism were significantly enriched in drones of Eastern bees, whereas lysosome and cell homeostasis were enriched in Western and African drones, respectively (supplemental Fig. S8).

A more detailed analysis of those proteins found in significantly higher abundances in larvae after mite exposure (supplemental Tables S22–S24) resulted in three findings: first, we found that—independently of genotype, male larvae always upregulated significantly fewer proteins in response to mite exposure compared with workers (generalized linear model, Wald Χ^2^ = 30.739, *p* < 0.001), indicating that worker larvae seem to responded in a more complex way compared with males. Second, we found remarkable little overlap between the groups of upregulated proteins, which was the case for both the genotype and caste comparisons (see supplemental Table S25 for details). This finding was in line with our overall proteomic and earlier GC–MS analyses of substantial differences in larval physiologies between castes and genotypes. Therefore, we concluded that immune-related molecular changes in response to mite infections are highly species and caste specific. Despite this, we found that a substantial number of upregulated proteins have well-established links to physiological processes that are linked to immune and/or stress responses (supplemental Table S24). In fact, immune and/or stress-related proteins were statistically overrepresented in our lists of upregulated proteins in workers of Eastern (Chi-square test, Χ^2^ = 19.1275, *p* < 0.001) and Western honey bees (Chi-square test, Χ^2^ = 4.6656, *p* < 0.031) as well as Western male larvae (Chi-square test, Χ^2^ = 21.7039, *p* < 0.001) when compared with our lists of total respective larval hemolymph proteomes (supplemental Tables S24 and S25 for statistical details).

**Fig. 9:**
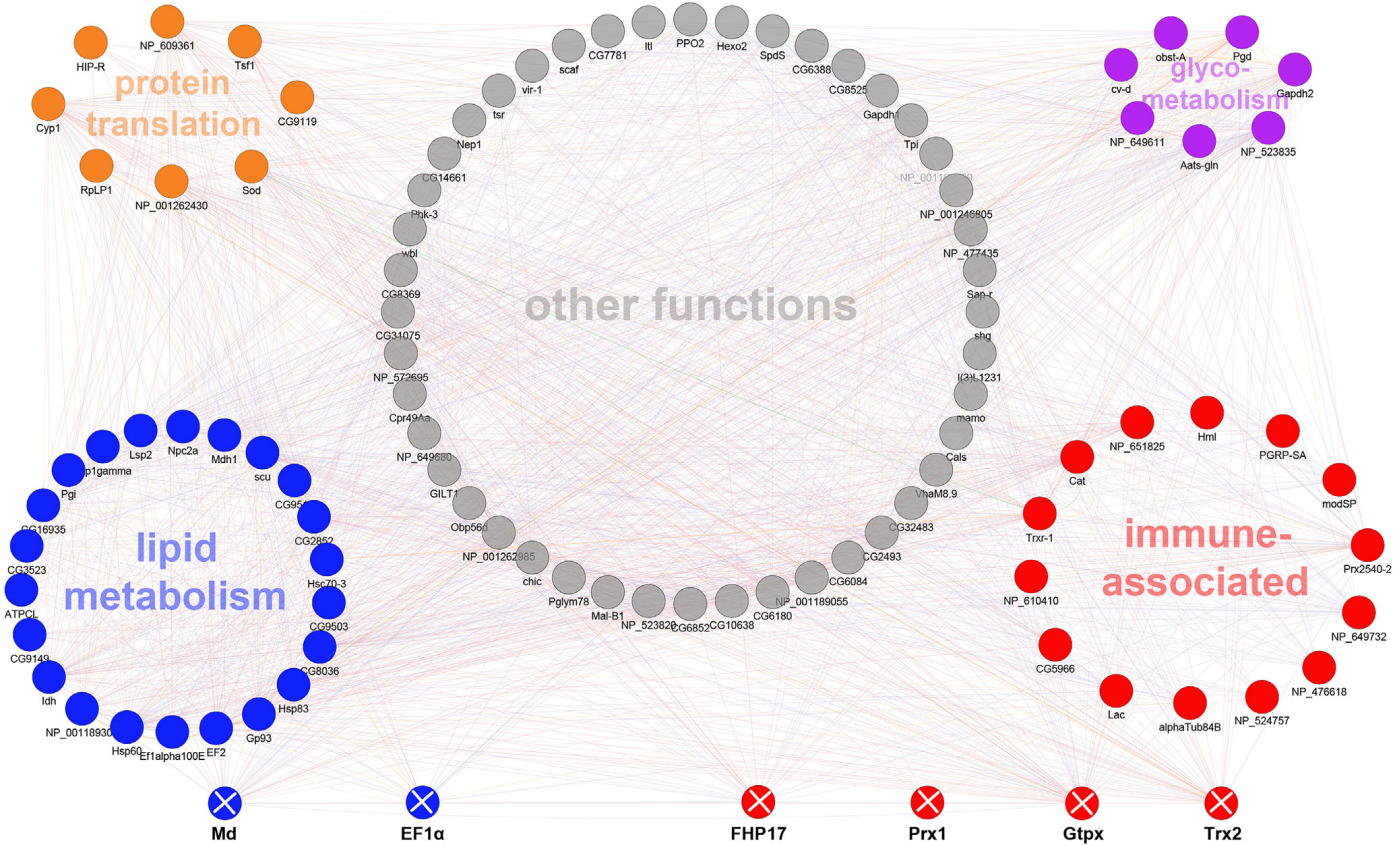
**Protein–protein interaction (PPI) network of the differentially expressed proteins among larval hemolymph of *Varroa* challengedEastern (*Apis cerana cerana*),Western(*Apis mellifera ligustica*), andAfrican(*Apis mellifera scutellata*)honey beeworkersand drones.** The PPI map was built using GeneMANIA against a *Drosophila* background. The node proteins were clustered according to their gene ontology (GO) annotation. The networks were visualized in Cystoscope. For node ID and more information, see supplemental Table S26. The proteins marked with an X were used for quantitative real-time PCR analysis, see Figure 10.

**Fig. 10:**
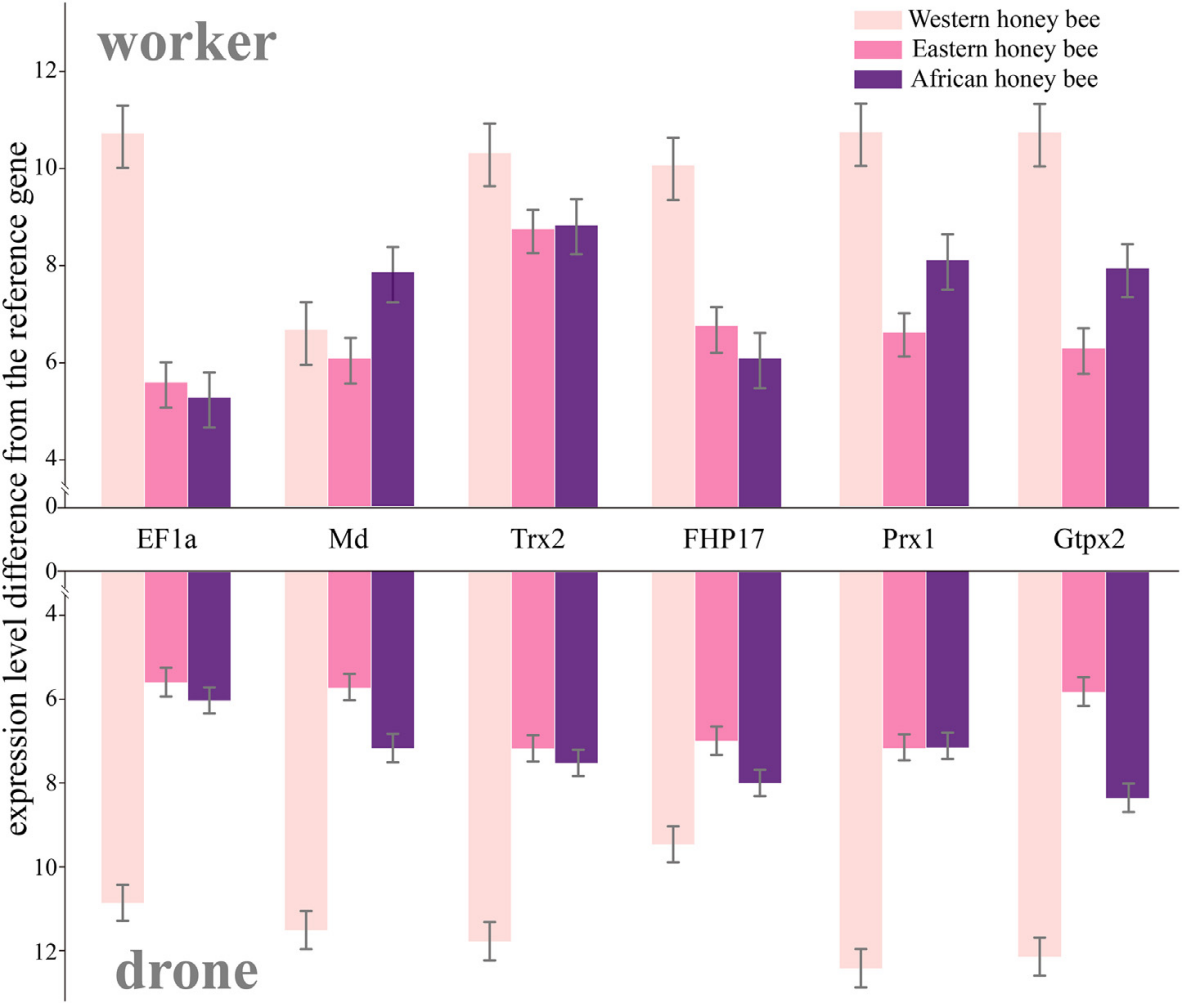
**Quantification of gene expression using real-time PCR offour immune-related(Trx2, FHPI17, Prx1,andGtpx2)and two non**-**immune-related (EF1α, Md)proteinsidentified in theprotein–protein interaction networkof *Varroa***-**challenged larvae (see**Fig. 9**).** The mRNA expression of each gene was normalized to the reference gene ribosome protein S18. Error bars depict standard deviations. Abbreviated protein names are as follows: EF1a (Elongation Factor 1-α), Md (Malat dehydrogenase), Trx2 (Thioredoxin - 2), FHP17 (Ferritin heavy polypeptide-17, Prx1 (Peroxiredoxin), and Gtpx2 (Phospholipid hydroperoxide glutathione peroxidase), see supplemental Table S1 for further details.

To identify a common set of proteins that distinguish susceptible from tolerant honey bee larvae, we used a PPI network to investigate those proteins that were differentially expressed in the three genotypes and two castes using the data of the *Varroa*-exposed larvae only (Fig. 9 and supplemental Table S26). Proteins involved in immune, translation, and lipid metabolism accounted for 18%, 7%, and 24% of all proteins, implying they play a crucial function in response to mite exposure. We consequently selected four immune-related node proteins from the PPI network and two additional proteins as controls to validate their protein expression differences on the transcriptional level using qRT–PCR (Fig. 10).

## Discussion

We conducted a series of behavioral and molecular experiments to unravel key interactions between the parasitic mite *V. destructor* and their larval honey bee hosts. We used a comparative approach and conducted our experiments using different bee genotypes and castes that are known to differ in their susceptibility to mite infections. Male larvae are generally assumed to be more susceptible and have higher mite infestation levels compared with worker larvae (53, 54). Furthermore, African and Eastern honey bees have been reported to have higher levels of mite tolerance compared with Western bees (26, 30). Our comparative approach therefore allowed us to narrow down on individual molecules or molecular networks that we assume to be part of the innate immune system of honey bees and that can be linked to increased or decreased levels of disease tolerance. Overall, we find that honey bees consistently respond to the presence of *Varroa* mites, but there was surprising variation in these innate immune responses between genotypes and castes. Further work will be required to understand how these proteome changes translate into host susceptibility or tolerance, and their effects on host and parasite fitness, but our findings imply that these interactions are highly complex and under strong natural selection.

We started our work with a behavioral assay to compare age-based host attractiveness of different honey bee larvae to *Varroa* mites. We found that, irrespectively of genotype or caste, mites were always most attracted to larvae at an age of 6 days, which coincides with the period when the brood cells are getting closed with a cap (55). From the parasite’s perspective, selecting their hosts shortly before capping provides an advantage if it reduces the opportunity for nurse bees to detect and remove mites or to minimize the time frame for larvae to detected mites and signal their presence to other colony members. We found no evidence that male larvae were generally more attractive to mites compared with workers during this initial phase of mite host choice. This implies that the documented differences in mite infestation rates and reproductive success between castes seem driven by interactions between hosts and parasites after these initial stages of infestation. Based on our findings, we concluded that larval social and/or innate immune responses seem key determinants of mite fitness and could explain documented differences in mite infestations between bee genotypes and castes. In order to further explore this idea, we conducted additional experiments and focused our remaining investigations on 6-day-old larvae, as they are most likely to become infected. Our study of volatile profiles of bee larvae that were not exposed to mites revealed that several volatiles could be used by mites during host choice but also revealed surprising variation between castes and genotypes. And even for the relatively small set of shared volatiles, we still found significant differences in their abundance between genotypes and castes. We found that the abundance of some of these volatiles are indeed age dependent and could therefore be used by mites to discriminate between larval hosts. Earlier work indicated that *Varroa* mites indeed use volatile cues to locate and select their larval hosts (56, 57). However, we found no consistent volatile pattern that separated susceptible bee castes and genotypes from more tolerant ones. Further investigations should therefore test whether larvae exposed to mites alter their volatile profiles, for example, to alarm nurse or hygienic bees about their infection status as part of a social immunity response. In general, a detailed understanding about these initial host–parasite interactions could be used in various ways for future mite management, either by specifically breeding less attractive bee larvae to slow down parasite propagation or to develop lures/baits that could be deployed into hives to collect mites.

In a next step, we tested for the possible presence of innate immune responses of honey bee larvae in response to mite infestations. We conducted a large-scale proteomic experiment and quantified differences in hemolymph proteomes in response to parasite exposure. We obtained and analyzed complete sets of hemolymph proteomes of 6-day-old male and worker larvae for all three honey bee genotypes, which is—to our knowledge—the largest comparative proteomic study in honey bees conducted so far. We found remarkably little overlap in these proteomes, both on the genotype as well as on the caste level, indicating that the developmental physiologies in different bees and castes differ substantially, even in closely related species such as African and Western honey bees. Although we were surprised by the amount of variation, we detected between these proteomes, both in composition and abundance, proteomic variation of similar scale has already been documented between honey bee larvae and imagoes or between different honey bee castes (46, 58). In so far, the differences we picked up were surprising but not necessarily unexpected based on available studies conducted earlier.

Despite this, we found that all larval groups studied responded with significant changes in their hemolymph in response to *Varroa* mite exposure, irrespectively of castes or genotype. We therefore concluded that larvae are able to recognize the presence of mites. The observed changes in proteomes can consequently be defined as complex immune responses in anticipation of mites starting their reproductive cycle and the expected damages caused. Similar to the overall proteomes, we found that the proteomic responses following parasite exposure differed substantially between castes and genotypes. When we looked at the proteomic responses of larvae in response to mite exposure, we found that upregulated proteins were linked to different biological functions such as immune defense and stress, supporting the idea of a targeted immune response following infection. We also noted that Eastern and Western honey bees were characterized by a significant enrichment of proteins with known links to immunocompetence. A similar pattern was found between castes where proteomes of worker larvae responded more strongly compared with male larvae. Our proteomic results could therefore explain why mite fitness is higher when the parasite develops on male compared with worker larvae. We therefore provide empirical evidence for the idea that differential reproductive success of mites seems determined by genetic differences in host immune responses, rather than mite host choice preferences which we did not find any evidence for in our behavioral experiments.

## Conclusion

In summary, we found that honey bee larvae are most susceptible to mite infestations shortly before brood cell capping, irrespectively of their genotype or castes. However, our findings imply that honey bee larvae recognize the presence of mites and alter the abundance of proteins linked to immune and stress responses. We found such responses to be fundamentally different in mite-tolerant bee larvae such as Eastern and African compared to Western honey bees. The identification of proteins and protein networks that are linked to mite tolerance offer multiple pathways for future research to understand how such larval immune responses impact mite fitness. Of particular interest for further study are those proteins and protein pathways, which we found to be upregulated in more mite-tolerant bees such as African and Eastern honey bees with well-documented links to immunity or stress responses (supplemental Table S25). Unraveling the exact mechanism how these proteins or protein networks increase mite tolerance offers unique opportunities to manage mites in the future, for example, by setting up molecularly informed breeding programs that focus on mite tolerance. For the latter, the study of Africanized honey bees might be of particular interest. These bees are a hybrid between African and Western honey bees and have been remarkably successful, given they were able to spread throughout South and Central America since they escaped from a laboratory in Brazil in 1957. They are also known for their increased levels of *Varroa* resistance (30, 59). Their ecological success implies that these bees inherited their tolerance traits from their African honey bee ancestors, something that could be studied in the future.

## Data Availability

All mass spectrometral data have been deposited to the ProteomeXchange Consortium (http://proteomecentral.proteomexchange.org) *via* the iProX partner repository (51) with the dataset identifier PXD029875.

## Supplemental data

This article contains supplemental data.

## Conflict of interest

The authors declare no competing interests.
