# Supplementary material for: Larval Exposure to Parasitic Varroa destructor Mites Triggers Specific Immune Responses in Different Honey Bee Castes and Species
Source: Mol Cell Proteomics. 2022 Jun 13;21(8):100257. doi: 10.1016/j.mcpro.2022.100257 (PMC9418504; doi:10.1016/j.mcpro.2022.100257)
Supplement: FigureS [file mmc1.pdf]

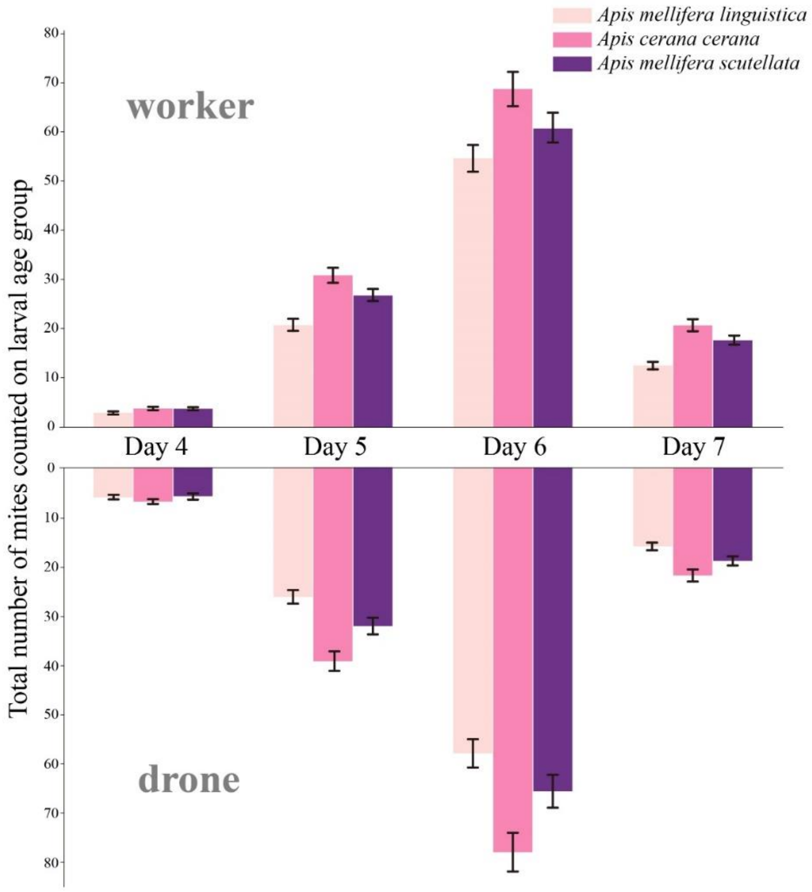

Figure S1. Residual mite loads in worker and drone of Eastern (*Apis cerana cerana,* Acc), Western (*Apis mellifera ligustica,* Aml) and African (*Apis mellifera scutellate,* Ams) honeybees at larval age day 4, 5, 6 and 7.

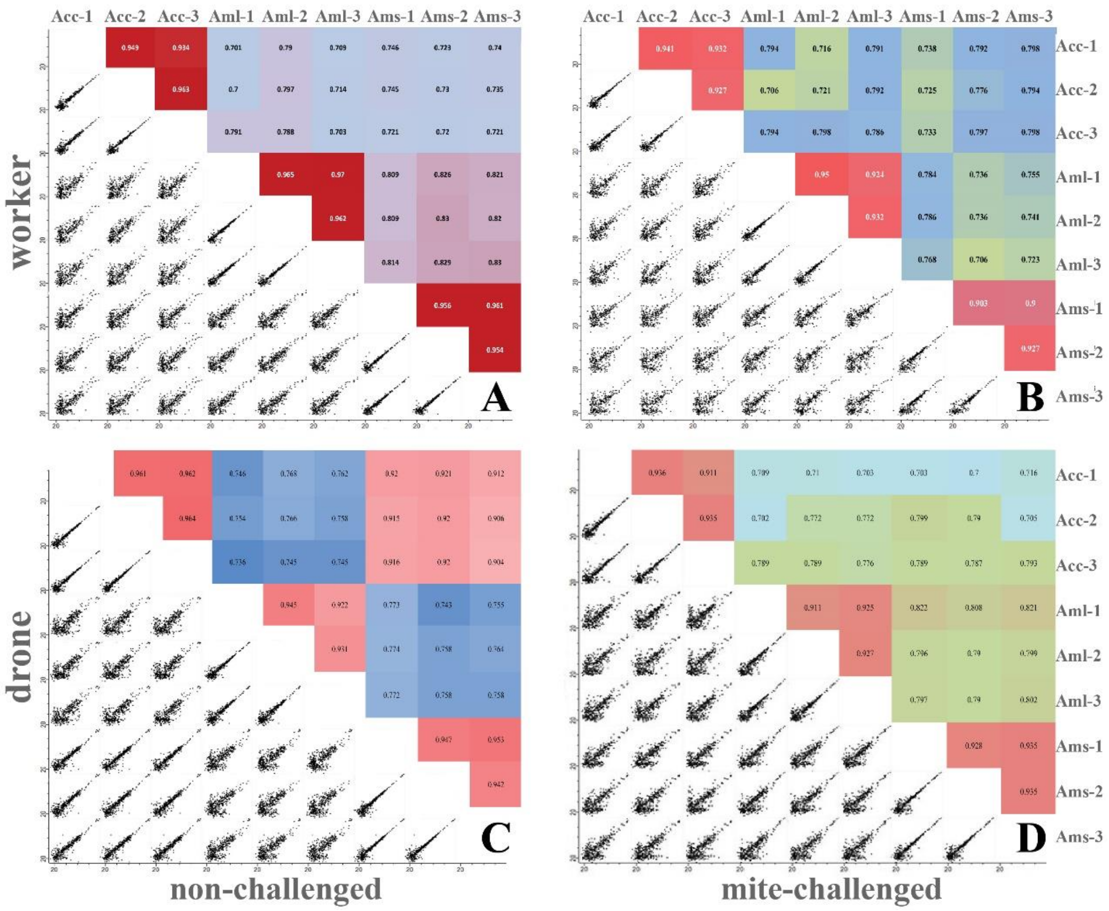

Figure S2. Overall Pearson's correlation coefficients in all the tested groups were from 0.9 to 0.97 between replicates of the same sample and varied from 0.7 to 0.921 among different samples.

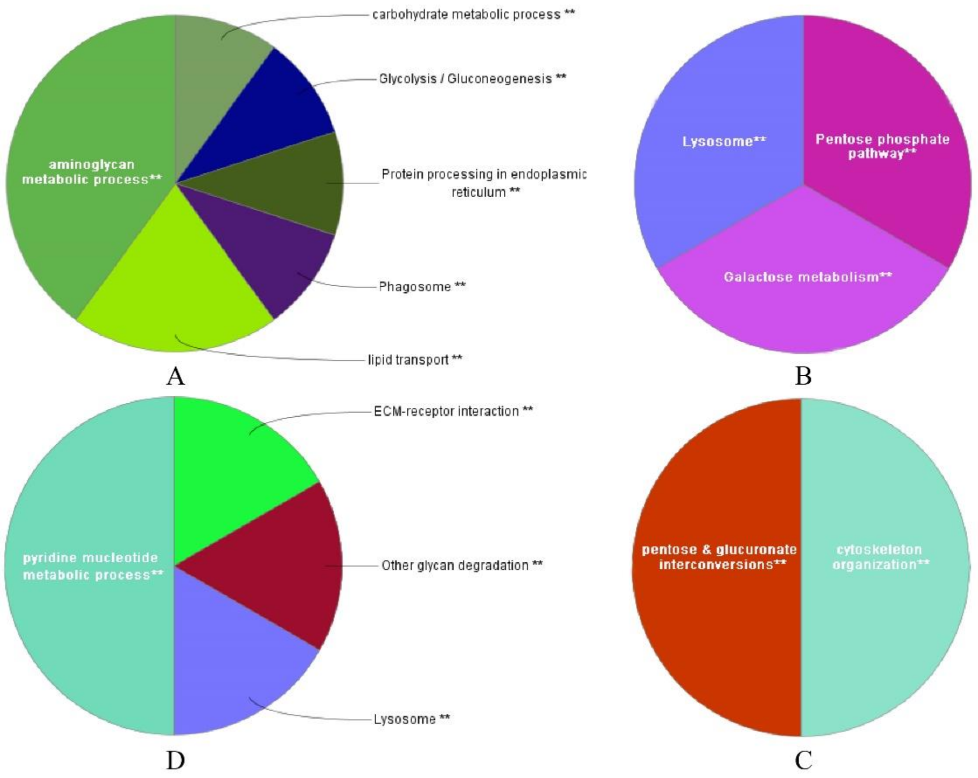

Figure S3. Qualitative comparison of identified hemolymph proteins *Varroa* non-challenged honeybee drone larvae of *Apis cerana cerana*, *Apis mellifera linguistica* and *Apis mellifera scutellata.* These identified proteins in the hemolymph of the three honeybee samples were analyzed using ClueGO to compare the functional classes and pathways specifically enriched by proteins at each of the samples. Pie chart A shows the significantly enriched functional classes and pathways shared among all the non-challenged honeybee samples. Moreover, the pie charts B, C and D indicate the specifically enriched functional classes and pathways in *Varroa* non-challenged *Apis cerana cerana*, *Apis mellifera linguistica* and *Apis mellifera scutellata* drones respectively. The double asterisks indicate significant enrichment at $p < 0.01$ statistical levels, respectively.

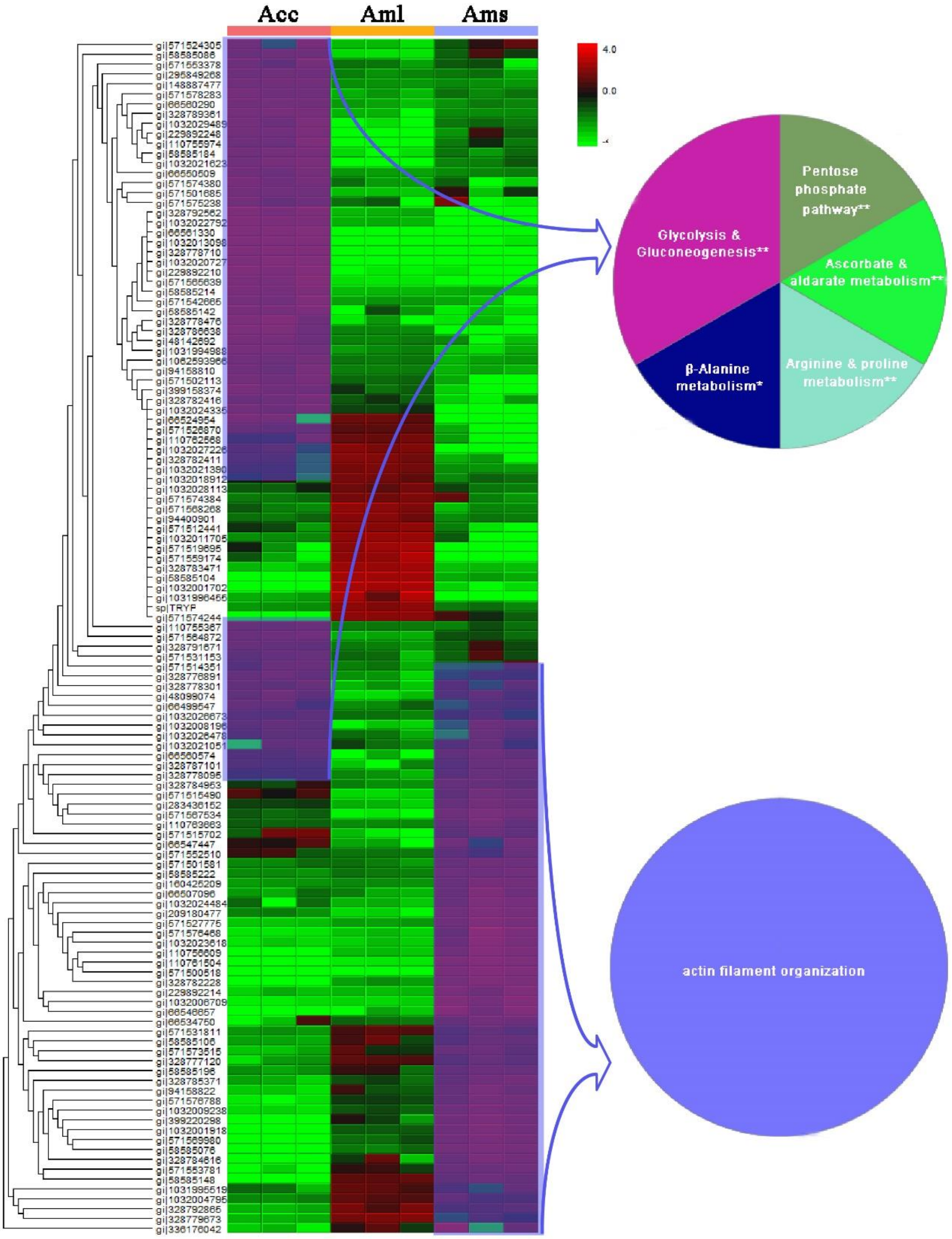

Figure S4. Hierarchical cluster and pathway enrichment analysis of the hemolymph quantitative comparison in non-challenged honeybee drone larvae of *Apis cerana cerana* (Acc), *Apis mellifera linguistica* (Aml) and *Apis mellifera scutellate* (Ams). Hierarchical cluster analysis was employed using the Perseus software v1.6.1 package. Cytoscape plug-in ClueGO v2.1.7 (http://apps.cytoscape.org/apps/cluego) was using for pathway enrichment analyses. This allowed us to test for significantly enriched KEGG pathways. To correct for multiple testing, we adjusted the significance levels using the Benjamini-Hochberg procedure.

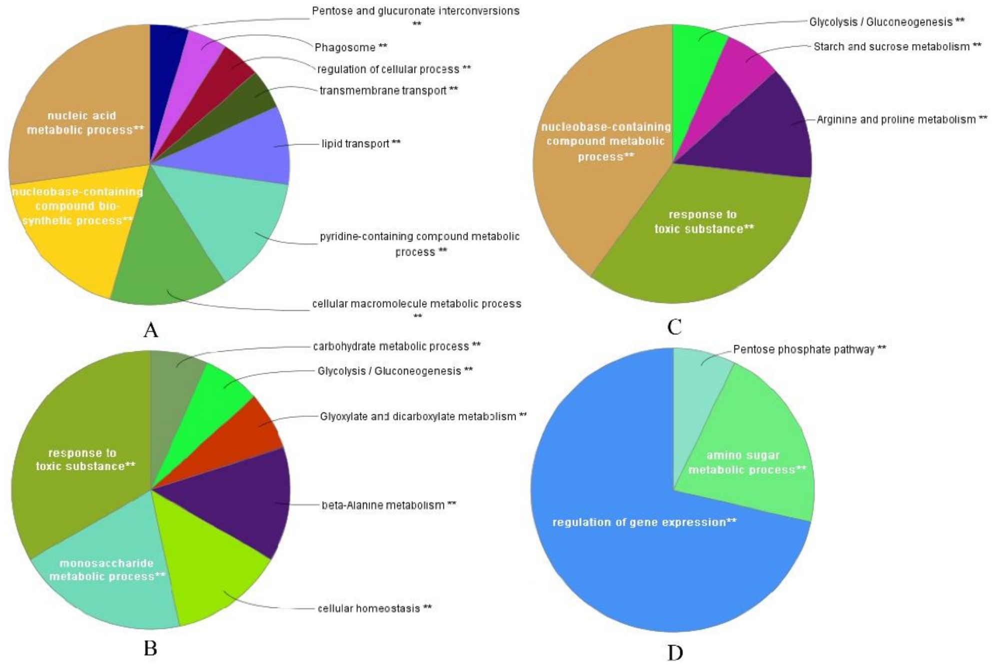

Figure S5. Qualitative comparison of identified hemolymph proteins *Varroa* challenged honeybee worker larvae of *Apis cerana cerana*, *Apis mellifera linguistica* and *Apis mellifera scutellata.* These identified proteins in the hemolymph of the three honeybee samples were analyzed using ClueGO to compare the functional classes and pathways specifically enriched by proteins at each of the samples. Pie chart A shows the significantly enriched functional classes and pathways shared among all the non-challenged honeybee samples. Moreover, the pie charts B, C and D indicate the specifically enriched functional classes and pathways in *Varroa* challenged *Apis cerana cerana*, *Apis mellifera linguistica* and *Apis mellifera scutellata* workers respectively. The double asterisks indicate significant enrichment at $p < 0.01$ statistical levels, respectively.

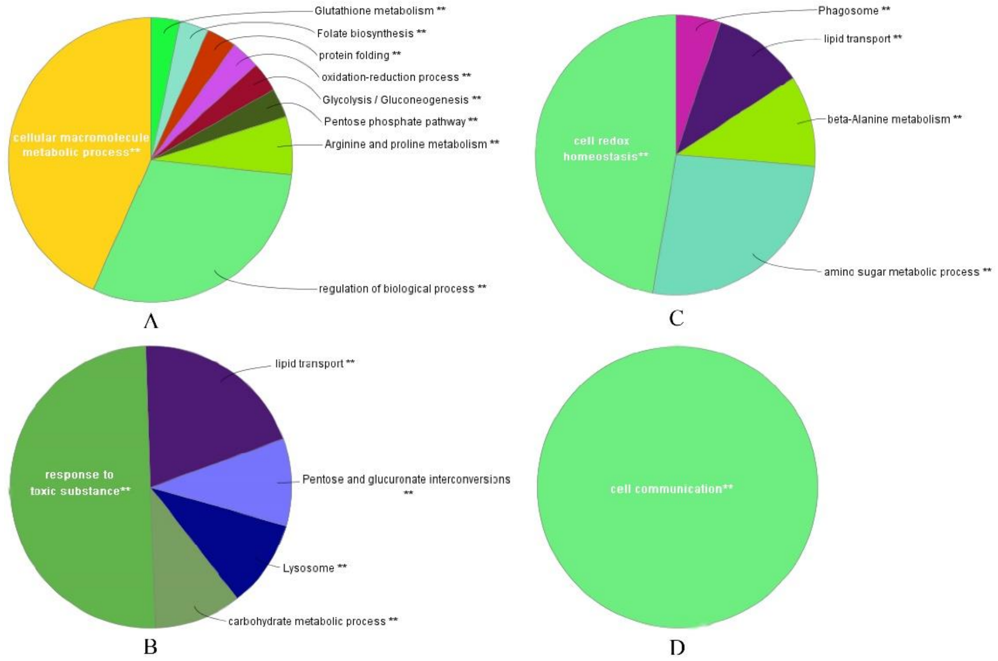

Figure S6. Qualitative comparison of identified hemolymph proteins *Varroa* challenged honeybee drone larvae of *Apis cerana cerana*, *Apis mellifera linguistica* and *Apis mellifera scutellata.* These identified proteins in the hemolymph of the three honeybee samples were analyzed using ClueGO to compare the functional classes and pathways specifically enriched by proteins at each of the samples. Pie chart A shows the significantly enriched functional classes and pathways shared among all the non-challenged honeybee samples. Moreover, the pie charts B, C and D indicate the specifically enriched functional classes and pathways in *Varroa* challenged *Apis cerana cerana*, *Apis mellifera linguistica* and *Apis mellifera scutellata* drones respectively. The double asterisks indicate significant enrichment at $p < 0.01$ statistical levels, respectively.

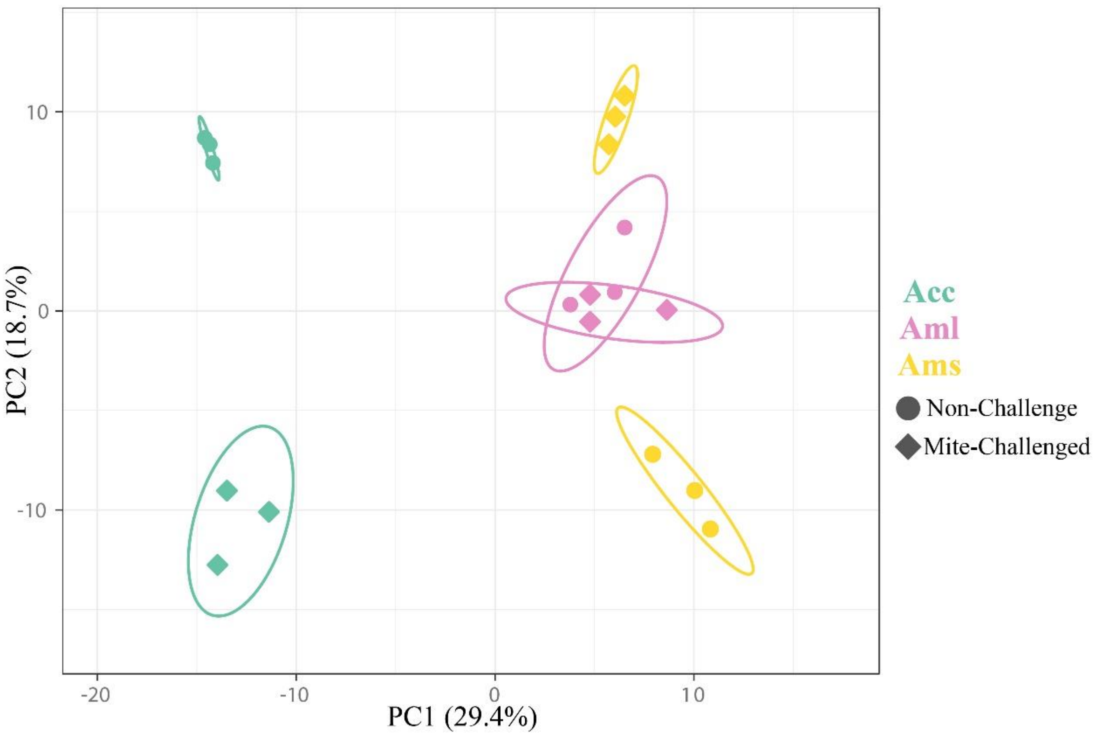

Figure S7. Principal component analysis of identified hemolymph proteins in *Varroa* non-challenged and challenged honeybee drone larvae of *Apis cerana cerana* (Acc), *Apis mellifera linguistica* (Aml) and *Apis mellifera scutellate* (Ams).

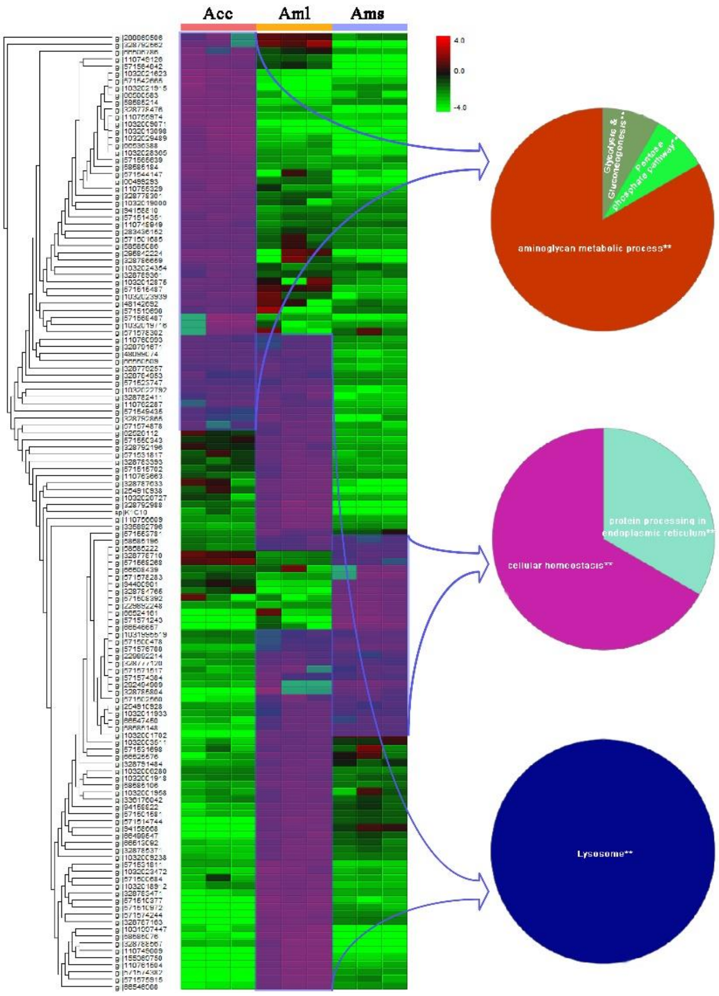

Figure S8. Hierarchical cluster and pathway enrichment analysis of the hemolymph quantitative comparison in mite-challenged honeybee drone larvae of *Apis cerana cerana* (Acc), *Apis mellifera linguistica* (Aml) and *Apis mellifera scutellate* (Ams). Hierarchical cluster analysis was employed using the Perseus software v1.6.1 package. Cytoscape plug-in ClueGO v2.1.7 (http://apps.cytoscape.org/apps/cluego) was using for pathway enrichment analyses. This allowed us to test for significantly enriched KEGG pathways. To correct for multiple testing, we adjusted the significance levels using the Benjamini-Hochberg procedure.
